# UGGT1 enhances enterovirus 71 pathogenicity by promoting viral RNA synthesis and viral replication

**DOI:** 10.1371/journal.ppat.1006375

**Published:** 2017-05-17

**Authors:** Peng-Nien Huang, Jia-Rong Jheng, Jamie J. Arnold, Jen-Ren Wang, Craig E. Cameron, Shin-Ru Shih

**Affiliations:** 1 Research Center for Emerging Viral Infections, College of Medicine, Chang Gung University, Taoyuan, Taiwan; 2 Department of Medical Biotechnology and Laboratory Science, College of Medicine, Chang Gung University, Taoyuan, Taiwan; 3 Graduate Institute of Biomedical Science, College of Medicine, Chang Gung University, Taoyuan, Taiwan; 4 Department of Biochemistry and Molecular Biology, Pennsylvania State University, University Park, PA, United States of America; 5 Department of Medical Laboratory Science and Biotechnology, National Cheng Kung University, Tainan, Taiwan; 6 Clinical Virology Laboratory, Department of Laboratory Medicine, Chang Gung Memorial Hospital, Taoyuan, Taiwan; University of California, Irvine, UNITED STATES

## Abstract

Positive-strand RNA virus infections can induce the stress-related unfolded protein response (UPR) in host cells. This study found that enterovirus A71 (EVA71) utilizes host UDP-glucose glycoprotein glucosyltransferase 1 (UGGT1), a key endoplasmic reticulum protein (ER) involved in UPR, to enhance viral replication and virulence. EVA71 forms replication complexes (RCs) on cellular membranes that contain a mix of host and viral proteins to facilitate viral replication, but the components and processes involved in the assembly and function of RCs are not fully understood. Using EVA71 as a model, this study found that host UGGT1 and viral 3D polymerase co-precipitate along with other factors on membranous replication complexes to enhance viral replication. Increased UGGT1 levels elevated viral growth rates, while viral pathogenicity was observed to be lower in heterozygous knockout mice (Uggt1 +/- mice). These findings provide important insight on the role of UPR and host UGGT1 in regulating RNA virus replication and pathogenicity.

## Author summary

Positive-strand RNA viruses are adept at hijacking host cell machinery to promote viral propagation, including the formation of RCs containing viral and host proteins on intracellular membranes to facilitate virion assembly and avoid detection by host defense mechanisms. However, the processes by which RCs are assembled, as well as the host proteins involved, have not been fully elucidated as yet. Here, we show that the endoplasmic reticulum (ER) protein UGGT1, a key regulator of the UPR host defense mechanism, co-precipitates with the 3D polymerase of EVA71 to facilitate RC formation, enhance viral RNA synthesis, and promote viral replication. Knockout of *Uggt1* reduced viral pathogenicity in animal studies. These findings highlight the role to which viruses can hijack key host proteins to promote viral replication, and may serve as the basis for the development of novel anti-viral strategies.

## Introduction

Positive-strand RNA viruses are capable of infecting a wide range of hosts, ranging from algae to humans. The mechanism underlying this broad range of pathogenicity spanning different hosts and tissue types involves the use of cellular membranes for viral genomic RNA replication, which provides a number of key benefits. Membrane structures allow buildup of a high local concentration of viral proteins, while also serving as a protective screen against protease cleavage. Membranes can further provide a structural scaffold that facilitates the correct spatial organization of viral replication complex (RC) components, and RCs can also be protected by the membrane against host infection sensors or other defense mechanisms [1,2]. Different positive-strand RNA viruses utilize different cellular membranes, resulting in a variety of morphological alterations; however, the sequences and functional domains of key viral proteins involved in membrane utilization are quite conserved among these viruses, suggesting that there are common strategies for the incorporation of cellular membranes into viral RCs [3,4].

Picornaviruses are a family of small positive-strand RNA viruses that include several notorious animal and human pathogens, such as rhinoviruses, Coxsackie viruses, foot and mouth disease virus, hepatitis A virus, and enterovirus A71 (EVA71). EVA71 typically causes hand, foot, and mouth disease (HFMD), which is generally regarded as a mild childhood illness [5]; however, not along after its initial isolation in California during 1969 [6], several deadly EVA71 epidemics occurred in the 1970s [7–9], and the virus has recently been associated with severe neurological complications, such as brain stem encephalitis and acute flaccid paralysis, in Asian infants and young children [10]. Several large HFMD outbreaks in the Asia-Pacific region have also occurred in recent years, including Malaysia, 2007 [11]; Taiwan, 1998 [12]; Singapore, 2000 [13]; Japan, 1997 and 2000 [14]; Shandong, China, 2007 [15]; and Fuyang, China, 2008 [16,17].

EVA71 genomic RNA is about 7,400 nucleotides (nt) long, and upon viral entry into host cells, the RNA genome is directly translated into one polyprotein, which is then cleaved by virus-specific proteases into structural and replication proteins. About 10 mature proteins and several other intermediate products are generated during this process, and these elements go on to perform many independent functions in the viral life cycle [18,19]. One non-structural protein that plays a key role in EVA71 replication is the 3D viral polymerase, which is encoded in the P3 viral genome region and is cleaved by viral proteases from the 3CD precursor proteinase after translation [20–22]. The 3D polymerase is an RNA-dependent RNA polymerase (RdRp) responsible for plus-strand and minus-strand viral RNA synthesis in viral RCs [23,24]. The first step in this process involves uridylylation of the small viral protein, VPg, in which two uridine monophosphate (UMP) molecules bind to the hydroxyl group of a tyrosine residue near the N-terminus of VPg via a reaction catalyzed by the viral 3D polymerase [25]. The 3D polymerase can also facilitate viral RNA chain elongation in viral RCs [26–29], and is known to interact with several host proteins, including Sam68 [30].

During picornavirus infection, viral RNA replication occurs on the cytoplasmic surfaces of single-membrane vesicles derived from the endoplasmic reticulum (ER), and the membranes can serve to accelerate RC assembly during positive-strand genomic RNA replication [31]. Viral proteins 2BC and 3A are known to be involved in viral RC formation, and these proteins contain hydrophobic domains that allow them to interact extensively with cellular membranes [32,33]. Viral protein 3A also plays an important role in membrane reorganization through its interactions with cellular proteins such as GBF1, Arf1, and PI4KIIIβ [34–37]. Other non-structural viral proteins are known to interfere with cellular membrane metabolism, and even rearrange subcellular organelles. Many viral and host proteins and lipids are involved in the membrane remodeling process induced by RCs, and the underlying mechanisms are complex and not well understood; for example, the 3D viral polymerase does not have obvious membrane binding sequences or properties, and its presence in RCs is therefore quite puzzling.

To enhance the current understanding of RC components and viral RNA replication following picornavirus infection, we used EVA71 as a model to evaluate interactions between the 3D viral polymerase and host proteins after virus infection. Proteins associated with 3D polymerase were immunoprecipitated with an anti-3D monoclonal antibody, and results showed that the host protein, UDP-glucose glycoprotein glucosyltransferase 1 (UGGT1), associates with 3D polymerase. UGGT1, also known as HUGT1, is a soluble ER protein that selectively reglucosylates unfolded glycoproteins, thus providing quality control for proteins transported out of the ER. Viral infections drive the accumulation of unfolded and misfolded proteins in the ER [38], and to reduce the adverse effects of such accumulation, the host cell utilizes a stress-related defense mechanism known as the unfolded protein response (UPR) to decrease the load of newly synthesized proteins within the ER and eliminate incorrectly folded proteins [39–41]. Alternatively, proteins possessing non-native structures are recognized by UGGT1, reglucosylated, and targeted for chaperone rebinding and ER retention [42]. UGGT1 can also add glucose molecules to the N-linked glycans of non-glucosylated substrates that fail quality control tests, thereby supporting additional rounds of chaperone binding and ER retention [42–46]. It has been shown that the disruption of protein folding in the ER induces UGGT1 expression [47]. Importantly, during EVA71 infection, we found that UGGT1 expression levels increase, and UGGT1 also redeploys from the ER to the cytoplasm, where it acts as a positive regulator of viral RNA synthesis. The 3A viral protein was also shown to increase UGGT1 and 3D polymerase levels in the membrane fraction. We further observed that the pathogenicity of EVA71 infection decreased in heterozygous *Uggt1* knockout mice. These findings shed light on the molecular processes driven by host UGGT1 and viral 3D protein association, and provide important insight on the relationship between virus pathogenicity and viral-host interactions.

## Results

### UGGT1 co-precipitates with viral 3D polymerase during EVA71 infection

**Fig 1:**
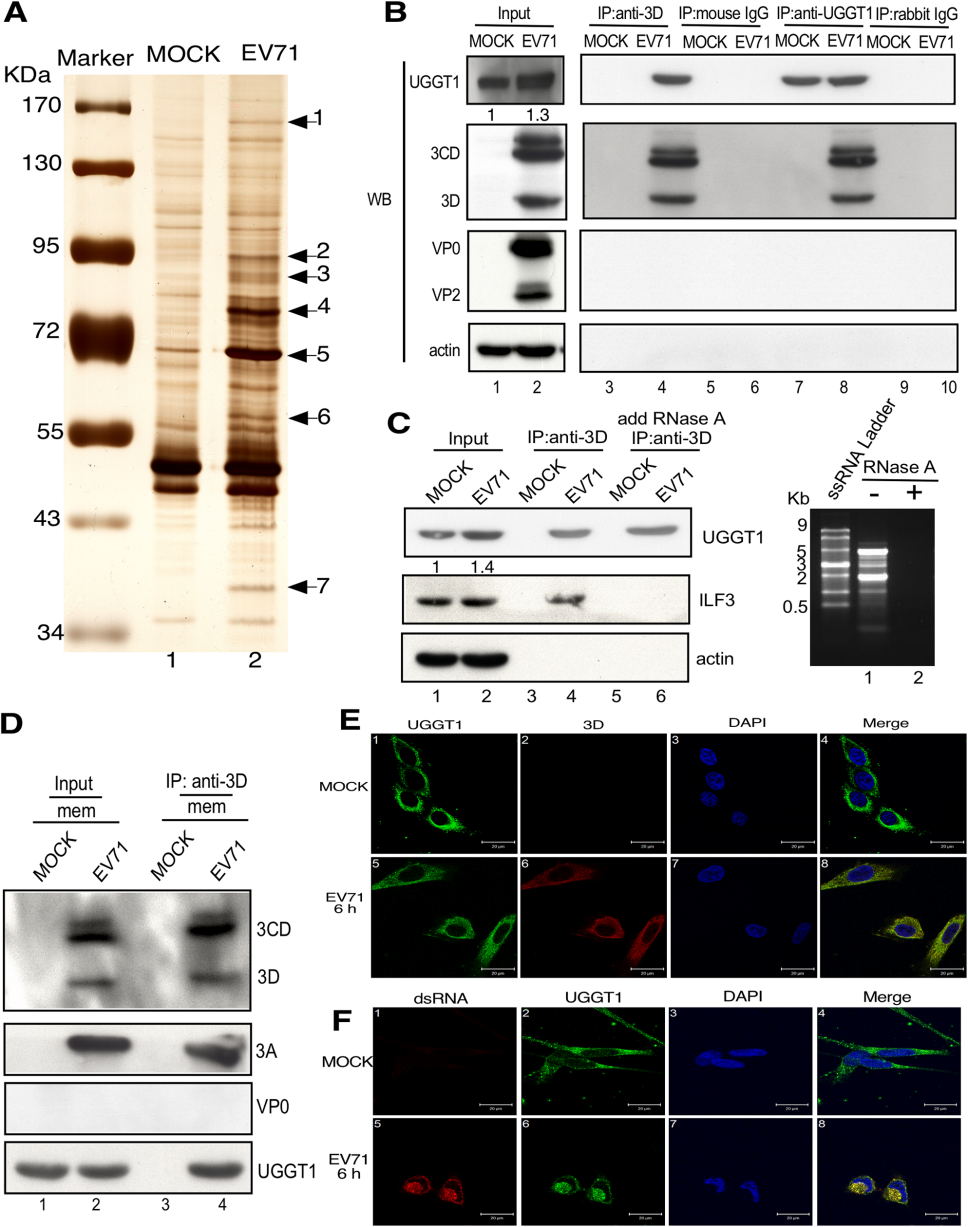
Co-precipitation of UGGT1 and the EVA71 3D viral polymerase. (A) At 6 hours post-infection, lysates from EVA71-infected or mock-infected cells were immunoprecipitated with anti-3D monoclonal antibody, and the precipitates were separated using SDS-PAGE, after which silver staining was applied for visualization. The seven labeled bands were excised, digested with trypsin, and analyzed by MALDI-TOF MS. (B) EVA71-infected and mock-infected cells were harvested and subjected to co-IP assays with anti-3D antibody (lanes 3 and 4) or mouse IgG (lanes 5 and 6); or anti-UGGT1 antibody (lanes 7 and 8) or rabbit IgG (lanes 9 and 10). The precipitates were analyzed using Western blotting with anti-UGGT1, anti-3D, anti-VP2, and anti-actin antibodies. (C) Cells were harvested at 6 h post-transfection, and lysates were treated with RNase A prior to being used in co-IP assays with an anti-3D antibody. Actin served as a loading control. Degradation of RNA was confirmed by RNA gel analysis. The precipitates were analyzed using Western blotting with anti-UGGT1, anti-ILF3, and anti-actin antibodies. (D) Membrane protein fractions were purified from EVA71-infected and mock-infected cells, and immunoprecipitation results with anti-3D antibody were analyzed by Western blotting with anti-3D, anti-3A, anti-VP2, and anti-UGGT1 antibodies. Expression of UGGT1, 3D, 3CD, 3AB, and 3A in the input lysate are shown. (E) EVA71-infected and mock-infected cells were fixed and stained with anti-UGGT1 and anti-3D antibodies at 6 h post-infection. An anti-UGGT1 antibody was used in panels 1 and 5, which were examined using a FITC filter. An anti-3D antibody was used in panels 2 and 6, which were examined using a rhodamine filter. Panels 3 and 7 display Hoechst 33258 staining results, and were examined using a 4’,6-diamidino-2-phenylindole (DAPI) filter. Panels 4 and 8 display merged rhodamine, FITC, and DAPI images. (F) EVA71-infected or mock-infected cells were fixed and stained with antibodies against UGGT1 and double strand RNA. Results with the anti-double strand RNA antibody are shown in panels 1 and 5, which were examined using a rhodamine filter. Anti-UGGT1 antibody was used for panels 2 and 6, which were examined using an FITC filter. Panels 3 and 7 display Hoechst 33258 staining results, which were examined using a DAPI filter. Panels 4 and 8 display merged rhodamine, FITC, and DAPI images.

To better understand how the EVA71 3D polymerase associates with host proteins and other components of the viral replication machinery at RCs, we sought to identify novel host factors that associate with the 3D polymerase in RCs. Accordingly, an anti-3D monoclonal antibody was used to perform immunoprecipitation assays, in order to purify proteins associating with the 3D polymerase in infected cells. We immunoprecipitated mock-infected and EVA71-infected cell lysates with this anti-3D monoclonal antibody, and subsequently identified seven major protein bands that appeared in the EVA71-infected lysates, but not in the mock-infected lysates (Fig 1A). We excised the protein bands that specifically associated with the 3D polymerase as shown in Fig 1A, digested the excisions with trypsin, and subjected them to matrix-assisted laser desorption/ionization time-of-flight mass spectrometry (MALDI-TOF MS) analysis. Table 1 presents these seven proteins and their accession numbers, as obtained from the US National Center for Biotechnology Information (NCBI) protein database (Table 1). The seven proteins included one viral protein, the EVA71 3CD polyprotein (Fig 1A, band 5), and six host proteins: UGGT1, elongation factor 2, interleukin enhancer binding factor 3 (ILF3), lamina-associated polypeptide 2 isoform alpha, T-complex protein 1 subunit theta, and eukaryotic translation initiation factor 3. The identified peptide sequences accounted for 24% of the UGGT1 sequence (Table 1 and S1 Fig). The ER protein, UGGT1 (Fig 1A, band 1), was selected for further investigation. We performed Western blot analysis to confirm that UGGT1 associated with the 3D polymerase following EVA71 infection. Co-IP experiments using EVA71-infected or mock-infected RD cell extracts were conducted, and the anti-3D antibody was able to immunoprecipitate UGGT1 (Fig 1B, lanes 3 and 4), while reciprocal co-IP experiments showed that the anti-UGGT1 antibody was also able to immunoprecipitate 3D polymerase in EVA71-infected cell lysates (Fig 1B, lanes 7 and 8). Viral capsid proteins were not observed in the UGGT1-viral protein complexes (Fig 1B). These results provide evidence that UGGT1 interacts with the EVA71 viral 3D polymerase.

**Table 1:** MALDI-TOF results of proteins associating with the EVA71 3D polymerase.

Spot No.	Protein Name	Match NCBI GI No.	Protein Score	Sequence Coverage	Mass (Da)
1	UDP-glucose glycoprotein glucosyltransferase 1	GI: 9910280	122	24%	177819
2	Elongation factor 2	GI: 4503483	185	46%	96246
3	Interleukin enhancer binding factor 3	GI: 212549553	88	47%	92144
4	Lamina-associated polypeptide 2, isoform alpha	GI: 1174689	70	39%	76016
5	Enterovirus 71 3CD	GI: 126567368	128	42%	73167
6	T-complex protein 1 subunit theta	GI: 48762932	88	51%	60153
7	Eukaryotic translation initiation factor 3	GI: 4503513	86	55%	36878

### EVA71 infection increases UGGT1 levels and interaction with 3D polymerase at RCs

**Fig 2:**
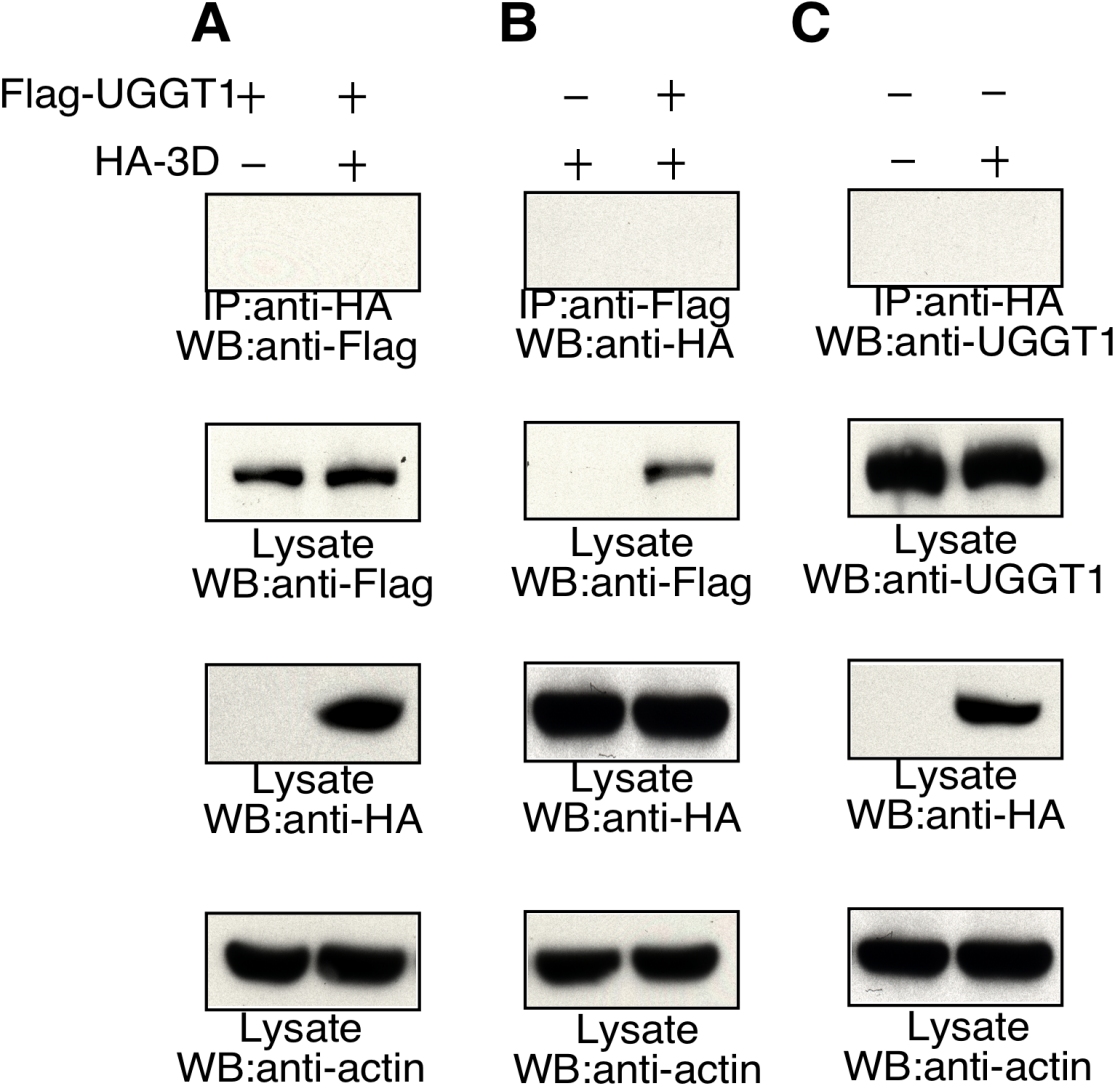
*In vivo* interaction between 3D polymerase and UGGT1. Uninfected RD cells were co-transfected with pFLAG-UGGT1 or pHA-3D DNA, and total cell lysates were harvested at 48 h after transfection for co-IP assays. (A) IP was performed with anti-HA, and the precipitates were analyzed by Western blotting (WB) with anti-FLAG. (B) IP was performed with anti-FLAG, and the precipitates were analyzed by WB with anti-HA. (C) Uninfected RD cells were transfected with vector or pHA-3D, and cell lysate proteins that were immunoprecipitated with anti-HA were subjected to WB with anti-UGGT1. The expression of FLAG-UGGT1 and HA-3D in the input lysates is indicated.

Host cells can mobilize the UPR in an attempt to restrict viral infection, and UGGT1 is known to be a key UPR factor in the ER. To ascertain UGGT1 expression levels in EVA71-infected cells, we compared UGGT1 levels in mock-infected and infected cells. UGGT1 expression levels were found to increase upon viral infection (Fig 1B and 1C, input lysate). It is known that the 3D viral polymerase associates with viral RNA, and to determine whether UGGT1 interaction with 3D polymerase was mediated by RNA, we examined the interaction between UGGT1 and viral genomic RNA. Treatment with RNase A prior to co-IP assays did not reduce UGGT1 interaction with the EVA71 3D polymerase, indicating that this interaction was not mediated by viral genomic RNA (Fig 1C, lanes 5 and 6). Host protein ILF3 is an RNA-binding protein that associates with the 3D viral polymerase (Table 1). Here, ILF3 served as a positive control for RNase A treatment. ILF3 association with the 3D viral polymerase was reduced after RNase A was applied prior to co-IP assays. Degradation of RNA was confirmed by RNA gel analysis (Fig 1C). To clarify the components of the UGGT1-3D complex, we purified the membrane fraction of EVA71-infected cells, and performed an immunoprecipitation assay to identify other viral proteins involved. The results shown in Fig 1D indicate that the 3A viral protein was also present in the UGGT1-3D complex, and this provides evidence to support an indirect interaction dependent on other viral proteins between UGGT1 and the 3D polymerase. In addition, as the 3D viral polymerase is located in the nucleus and cytoplasm at different stages of viral replication, while UGGT1 is located predominantly in the ER, we therefore sought to examine how UGGT1 and the 3D polymerase colocalize intracellularly during EVA71 infection, using fluorescence confocal microscopy. In mock-infected cells, UGGT1 was predominantly localized in the cytoplasm (Fig 1E, panel 4), while in EVA71-infected cells, both the 3D polymerase and UGGT1 were localized in the cytoplasm at 6 h post-infection (Fig 1E, panel 8). Moreover, when an anti-dsRNA antibody was used to highlight the location of RCs in an immunofluorescence assay, staining results showed that UGGT1 associates with RCs in the cytoplasm (Fig 1F). However, co-IP experiments conducted in uninfected cells co-expressing Flag-UGGT1 and HA-3D showed that anti-HA antibodies did not precipitate Flag-UGGT1, nor did anti-Flag antibodies precipitate HA-3D. Anti-HA antibodies also did not co-immunoprecipitate endogenous UGGT1 from RD cells expressing HA-3D (Fig 2). These results show that UGGT1 can co-precipitate with EVA71 viral polymerases in RCs, and further indicate that viral infection is essential for UGGT1 co-purification with the EVA71 3D polymerase. Together, these findings confirm that upon viral infection, UGGT1 levels increase, and UGGT1 co-precipitates with 3D polymerase and other factors on membranous replication complexes.

### UGGT1 is a positive regulator of viral replication and propagation

**Fig 3:**
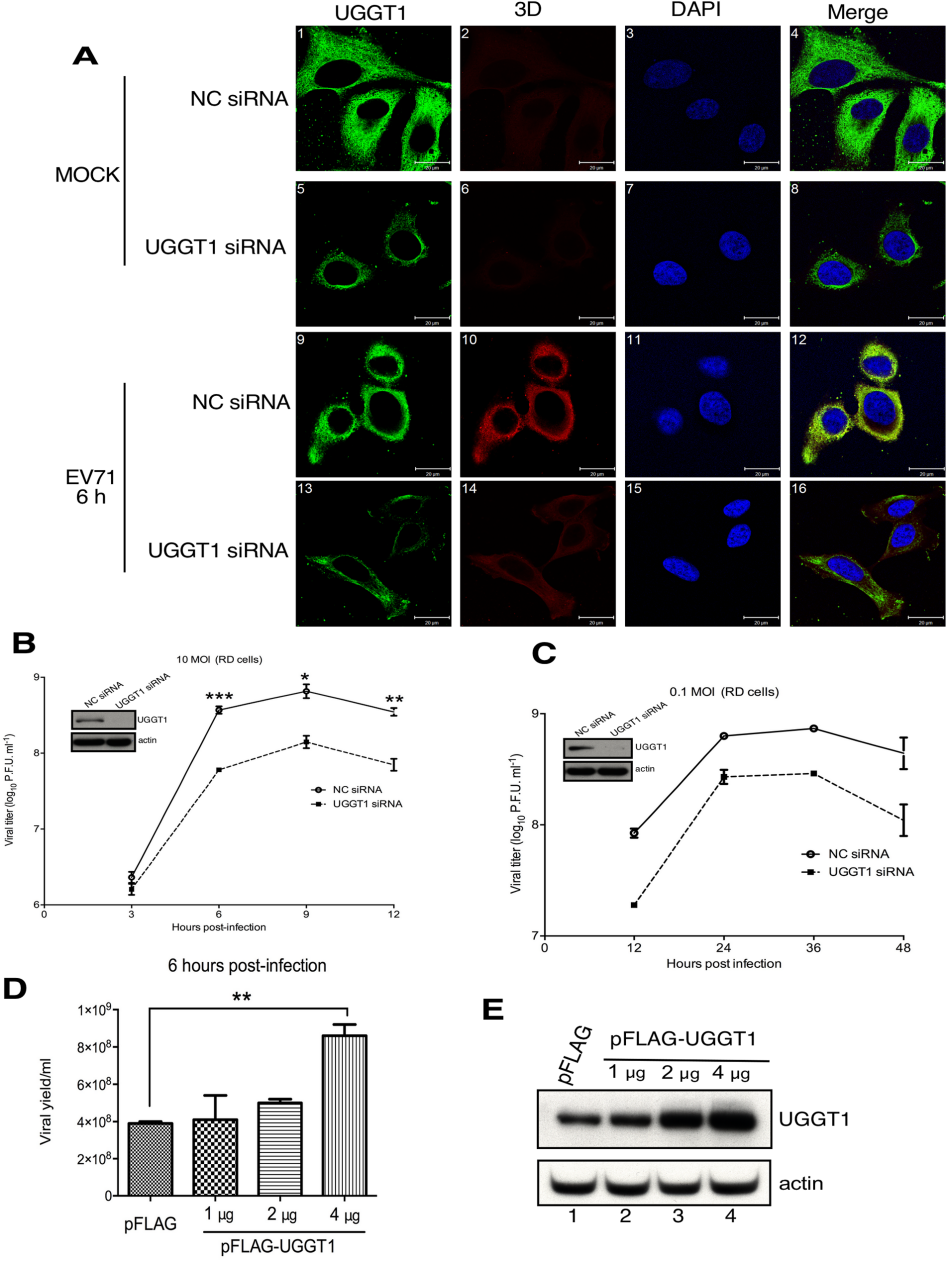
UGGT1 facilitates EVA71 replication and propagation. (A) Confocal microscopy results of UGGT1 and 3D expression in NC or UGGT1 siRNA-treated RD cells that were infected with EVA71 and subjected to immunostaining at 6 h post-infection. Panels 1, 5, 9, and 13 were stained with anti-UGGT1 and examined using a FITC filter; panels 2, 6, 10, and 14 were stained with anti-3D and examined with a rhodamine filter; and panels 3, 7, 11, and 15 were subjected to Hoechst 33258 staining and examined with a DAPI filter. (B) and (C) RD cells were transfected with NC or UGGT1 siRNA for 48 h, and then challenged with EVA71 at an MOI of 10 or 0.1. A plaque assay was performed to measure viral propagation rates at various timepoints post-infection. The left panels show the knockdown of uggt1 following siRNA treatment. (D) RD cells were transfected with 1, 2, or 4 μg of pFLAG-UGGT1 or pFLAG-vector for 48 h, and then challenged with EVA71 at an MOI of 10. A plaque assay was performed to measure viral yields at 6 h post-infection. (E) UGGT1 was overexpressed by respectively transfecting 1, 2, or 4 μg of plasmid pFLAG-UGGT1 to RD cells, and the panels show the corresponding increase in UGGT1 levels following overexpression, with actin serving as a loading control. ***P < 0.001, **P < 0.01, and *P < 0.05, as calculated by two-tailed unpaired Student’s t-test.

To ascertain the effect of UGGT1 on viral replication and propagation, we infected (negative control) NC or UGGT1 siRNA-transfected cells with a high titer of EVA71 (MOI = 10), and assessed 3D polymerase expression at 6 h post-infection by confocal microscopy. We first analyzed the effect of UGGT1 knockdown on siRNA-transfected cell viability. Cell viability was measured by a CellTiter-Glo Luminescent Cell Viability kit (Promega), which quantitates the ATP generated in viable cells. The results presented in S2 Fig demonstrate that cell proliferation and viability were not significantly different between NC siRNA- and UGGT1 siRNA-transfected cells (S2 Fig). In UGGT1 siRNA-treated cells, viral protein expression was significantly lower compared to NC siRNA-treated cells (Fig 3A, panels 10 and 14). These results suggest that UGGT1 plays a critical role in enhancing viral replication. To further evaluate the effects of UGGT1 on EVA71 replication rates, we treated RD cells with NC or UGGT1 siRNA, and then infected these cells with a high (MOI = 10) or low (MOI = 0.1) EVA71 titer. The plaque assay was used to detect viral yields at various timepoints post-infection. Viral replication rates were found to be lower in *uggt1* knockdown cells as compared to NC siRNA-treated cells, regardless of MOI levels (Fig 3B and 3C). These results support the hypothesis that UGGT1 is a positive regulator during EVA71 infection. We repeated this experiment in SF268 glioblastoma (SF268) cells to determine whether the effects of UGGT1 on viral replication are specific to a given cell type. Results showed that viral replication rates were also lower in UGGT1 siRNA-treated SF268 cells, as compared to NC siRNA-treated cells (S3A and S3B Fig). To avoid siRNA off-target effects, we used EVA71 to infect cells overexpressing UGGT1, and subsequently measured virus yields at 4, 6, and 8 h post-infection. The results showed that viral replication increased when UGGT1 was overexpressed in infected cells (Fig 3D, S3C and S3D Fig).

We further evaluated the role of UGGT1 in enterovirus D68 (EVD68). EVD68 is classified in a different group as other more common enteroviruses, such as EVA71 and coxsackievirus A16, but this does not mean it is less pathogenic: in a 2014 outbreak of EVD68, a total of 1,152 people in United States were confirmed as having acute respiratory infections caused by EVD68. It is important for emergency clinicians to recognize this viral illness, because it can lead to respiratory distress that requires hospitalization or, in some instances, intensive care [48]. After infecting NC or UGGT1 siRNA-treated cells with EVD68, we evaluated viral replication rates, and observed that EVD68 viral titers were lower in UGGT1 siRNA-transfected cells as compared to NC siRNA-transfected cells (S3E and S3F Fig). These results confirm that UGGT1 is a positive regulator of EVA71 and EVD68 replication, and suggest that it may be a commonly utilized host factor for viral replication in enteroviruses.

To assess whether the enzymatic activity of UGGT1 is critical for viral replication, we generated UGGT1 variants lacking monoglucosylation activity (UGGT1(mut)). Previously, it was shown that UGGT1 enzymatic activity would be abolished after the elimination of monoglucosylation activity via mutation [47]. We overexpressed UGGT1 or UGGT1(mut) in infected cells, and subsequent comparison of viral yields showed no significant difference (S4 Fig), suggesting that UGGT1 enzymatic activity is not critical for viral replication. In light of this, we propose that UGGT1 may primarily act as a protein bridge to facilitate viral replication.

### EVA71-induced pathogenicity decreases in *Uggt1* heterozygous knockout mice

**Fig 4:**
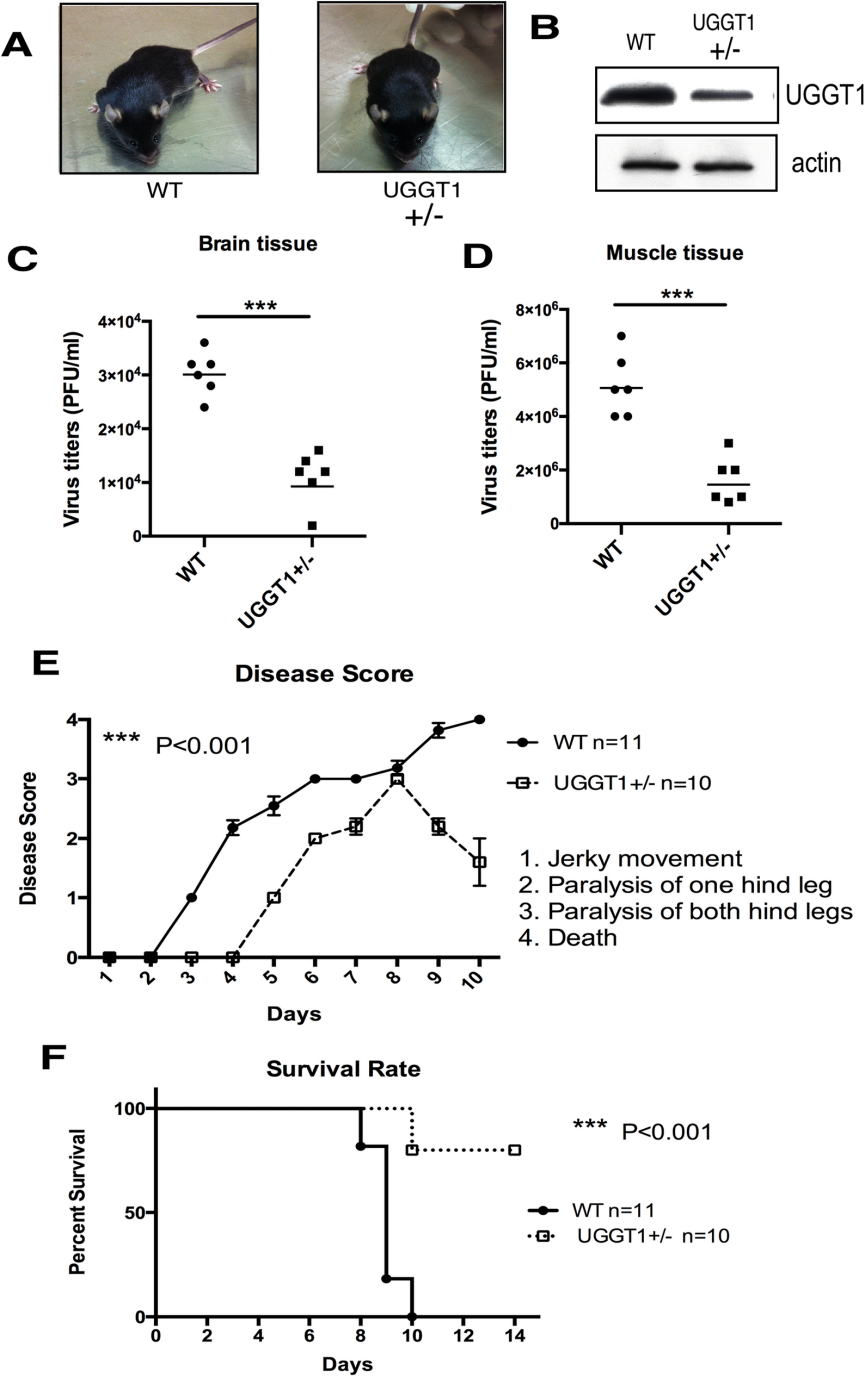
Viral yield, neurological symptoms, and lethality in UGGT1 heterozygous knockout mice infected with EVA71. (A) Phenotypes of UGGT1+/+ wild-type (WT) mice and UGGT1+/− heterozygous knockout mice. (B) UGGT1 expression levels in brain tissue homogenates of UGGT1+/+ WT mice and UGGT1+/− heterozygous knockout mice, as detected by western blotting. (C) and (D) 10-day-old WT or Uggt1 heterozygous knockout mice were injected with 10^5^ PFU/mouse of EVA71 strain MP4, and on Day 3 after infection, EVA71 virus was extracted from brain and muscle tissues and quantitated. (E) CNS-like hind limb paralysis and (F) Survival rates in 10-day-old WT and Uggt1 heterozygous knockout mice injected with 10^5^ PFU/mouse of the EVA71 MP4 strain were evaluated, and one-way ANOVA on ranks (Kruskal-Wallis H test) was used to determine statistical significance. The number (n) of mice in each group is shown.

To assess the role of UGGT1 in viral pathogenicity *in vivo*, we generated *Uggt1* knockout mice from the KOMP-CSD ES cell resource [43]. The *Uggt1* gene deletion destroys reglucosylation activity in cells and is embryonically lethal at day E13 in mice [45]. Homozygous *Uggt1* knockout mice were embryonically lethal; however, heterozygous mice were viable, fertile, developed normally, and did not reveal any obvious phenotypic alterations up to adulthood (Fig 4A). Heterozygous *Uggt1* knockout mice expressed only 50–60% of UGGT1 as compared to their UGGT1 wild-type littermates (Fig 4B). A mouse-adapted EVA71 strain with increased virulence in mice, MP4, was generated after four serial passages of the parental EVA71 strain 4643 in mice [46]. To quantify EVA71 replication rates in wild-type or heterozygous *Uggt1* knockout mice, the viral load in different mouse tissues on Day 3 after EVA71 infection was assessed. EVA71 was detected in the brain (Fig 4C) and muscle tissues (Fig 4D), but the viral load in *Uggt1* heterozygous knockout mice was significantly lower than that in wild-type mice. These results prompted us to investigate the virulence of EVA71 in *Uggt1* heterozygous knockout mice. We challenged 10-day-old wild-type or heterozygous *Uggt1* knockout mice with a 10^5^ plaque-forming unit (PFU)/mouse dose of EVA71 strain MP4. Wild-type mice displayed severe limb paralysis on Day 4 after infection, while heterozygous knockout mice only demonstrated mild limb paralysis (Fig 4E). Infected wild-type mice began to die on Day 8 after infection, whereas heterozygous *Uggt1* knockout mice began to die on Day 10; however, the 90% survival rate in infected heterozygous knockout mice was still significantly higher (*P* < 0.001) than the 0% survival in wild-type mice (Fig 4F).

To further ascertain if UGGT1 plays a similarly important role in other virus families with regard to enhancing virulence and pathogenicity, we selected the Japanese Encephalitis Virus (JEV) from the family Flaviviridae to study the role of UGGT1 upon virus infection. First, we performed Western blot analysis to confirm the association between UGGT1 and the NS5 polymerase following JEV infection. Immunoprecipitation experiments using JEV-infected or mock-infected BHK-21 cell extracts were conducted, and the anti-UGGT1 antibody was able to immunoprecipitate NS5 polymerase only in JEV-infected cell lysate (S5A Fig). To determine growth efficiency of the virus in mouse brains, an experiment was carried out in suckling mice by intracranial inoculation with 10^4^ PFU/mouse of the T1P1 JEV strain. After 7 days post-infection, we collected suckling mice brain tissue, and performed a plaque assay to determine viral titers. The results indicated that UGGT1 was able to associate with JEV polymerase NS5 and enhance viral growth efficiency in suckling mice tissue (S5B Fig). These results show that UGGT1 knockdown can reduce EVA71 and JEV virulence and improve disease outcome.

### UGGT1 enhances viral positive- and negative-strand RNA synthesis

**Fig 5:**
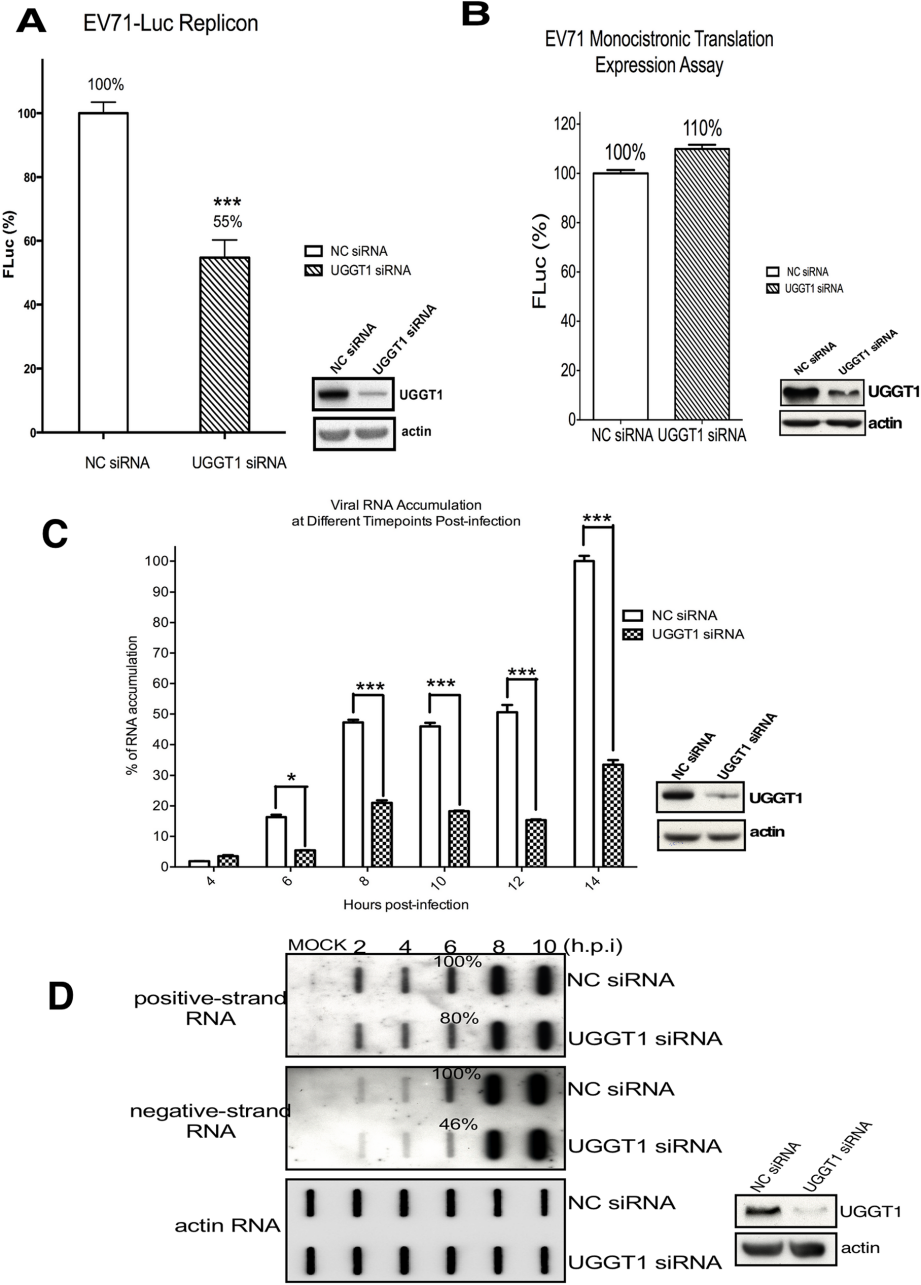
UGGT1 enhances viral RNA replication. (A) NC or UGGT1 siRNA-treated RD cells were transfected with EVA71-Luc replicon RNA, and cells were assayed for firefly luciferase signals (FLuc) at 6 h post-transfection. The right panel indicates the knockdown efficiency of Uggt1. (B) Monocistronic mRNA containing EVA71 IRES and FLuc was transfected to cells pretreated with NC or UGGT1 siRNA. At 6 h post-transfection, cell lysates were assayed for FLuc activity. Western blotting data indicates siRNA knockdown efficiency. Experiments were performed in triplicate to obtain the bar graph. (C) NC or UGGT1 siRNA-treated RD cells were infected with EVA71 at an MOI of 10. Intracellular viral RNA was isolated at 4, 6, 8, 10, 12, and 14 h post-infection, and quantitated using real-time RT-PCR. The amount of viral RNA at 14 h post-infection in NC siRNA-transfected cells was taken as 100%, and the relative amount of viral RNA isolated at each timepoint is presented as a percentage of this. The right panel indicates knockdown efficiency of Uggt1. (D) RD cells were transfected with NC or UGGT1 siRNA for 48 h and then reseeded. After 24 h, cells were infected with EVA71 at an MOI of 10, and RNA was extracted at 2, 4, 6, 8, and 10 h post-infection. RNA was loaded onto a nitrocellulose sheet in the slot blot manifold. The right panel demonstrates Uggt1 knockdown efficiency. ***P < 0.001 and *P < 0.05, as calculated by two-tailed unpaired Student’s t-test.

The EVA71 life cycle comprises entry, viral mRNA translation, viral RNA synthesis, and virus assembly. To evaluate the biological significance of UGGT1 in EVA71 replication, we examined the effects of UGGT1 on EVA71 replication efficiency. NC and UGGT1 siRNA knockdown RD cells were transfected with EVA71-Luc replicon RNA, and cell firefly luciferase activity (measured in relative light units, RLU) was measured at 6 h post-transfection. In EVA71-Luc replicon RNA, the viral genome P1 region was replaced with a firefly luciferase reporter gene, and luciferase expression therefore reflected viral replication. In UGGT1 siRNA-treated cells, EVA71-Luc replicon luciferase activity was reduced to 55% of the activity in control cells (Fig 5A). This could be due to loss of UGGT1 promotion of either viral mRNA translation or viral RNA replication. We therefore examined the effect of UGGT1 on EVA71 cap-independent translation first, using dicistronic and monocistronic IRES-mediated translation assays [49]. In the dicistronic translation assay, the first cistron (Renilla luciferase, RLuc) involved cap-dependent translation, while the second cistron (Firefly luciferase, FLuc) required EVA71 IRES-dependent translation. The ratio of FLuc expression to RLuc expression reflects IRES-mediated translation activity. We transfected RD cells with NC or UGGT1 siRNA, and a dicistronic reporter plasmid was then co-transfected. After 48 h post-transfection, cell lysates were collected and used to calculate the ratio of FLuc to RLuc. The results showed that dicistronic IRES activity in NC siRNA-treated cells was not significantly superior to the activity in UGGT1 siRNA-treated cells (S6 Fig), and monocistronic IRES activity also showed no significant difference between NC and UGGT1 siRNA-treated cells (Fig 5B). These results indicate that assisting viral mRNA translation is not the role played by UGGT1 in EVA71 infection.

As UGGT1 can be co-purified with the 3D polymerase, we speculated that UGGT1 may facilitate EVA71 replication by enhancing viral RNA synthesis. To ascertain this, we first monitored viral RNA production in NC or UGGT1 siRNA-treated RD cells that were subsequently infected with EVA71. Intracellular RNA was isolated at different intervals post-infection, and EVA71 viral RNA was measured using quantitative real-time reverse transcription polymerase chain reaction (RT-PCR). Results showed that viral RNA production was 33% lower in UGGT1 siRNA-treated cells, as compared to NC siRNA-transfected cells (Fig 5C). Viral RNA levels were further investigated in *Uggt1* knockdown cells. EVA71 was used to infect cells, and viral RNA was extracted at various timepoints post-infection. Slot blot analysis, using specific RNA probes that recognize positive or negative sense EVA71 RNA, was used to monitor viral RNA synthesis. The results in Fig 5D show that levels of both positive and negative sense EVA71 RNA were lower in UGGT1 siRNA-treated cells than NC siRNA-treated cells; specifically, positive-strand RNA levels were reduced by 20% in UGGT1 siRNA-treated cells, while negative-strand RNA levels were reduced by 54% (Fig 5D). These results suggest that UGGT1 likely acts to enhance viral RNA synthesis during EVA71 infection.

### UGGT1 deploys to the cytosol and colocalizes with viral proteins 3D, 3A, and 3AB in the membrane fraction

**Fig 6:**
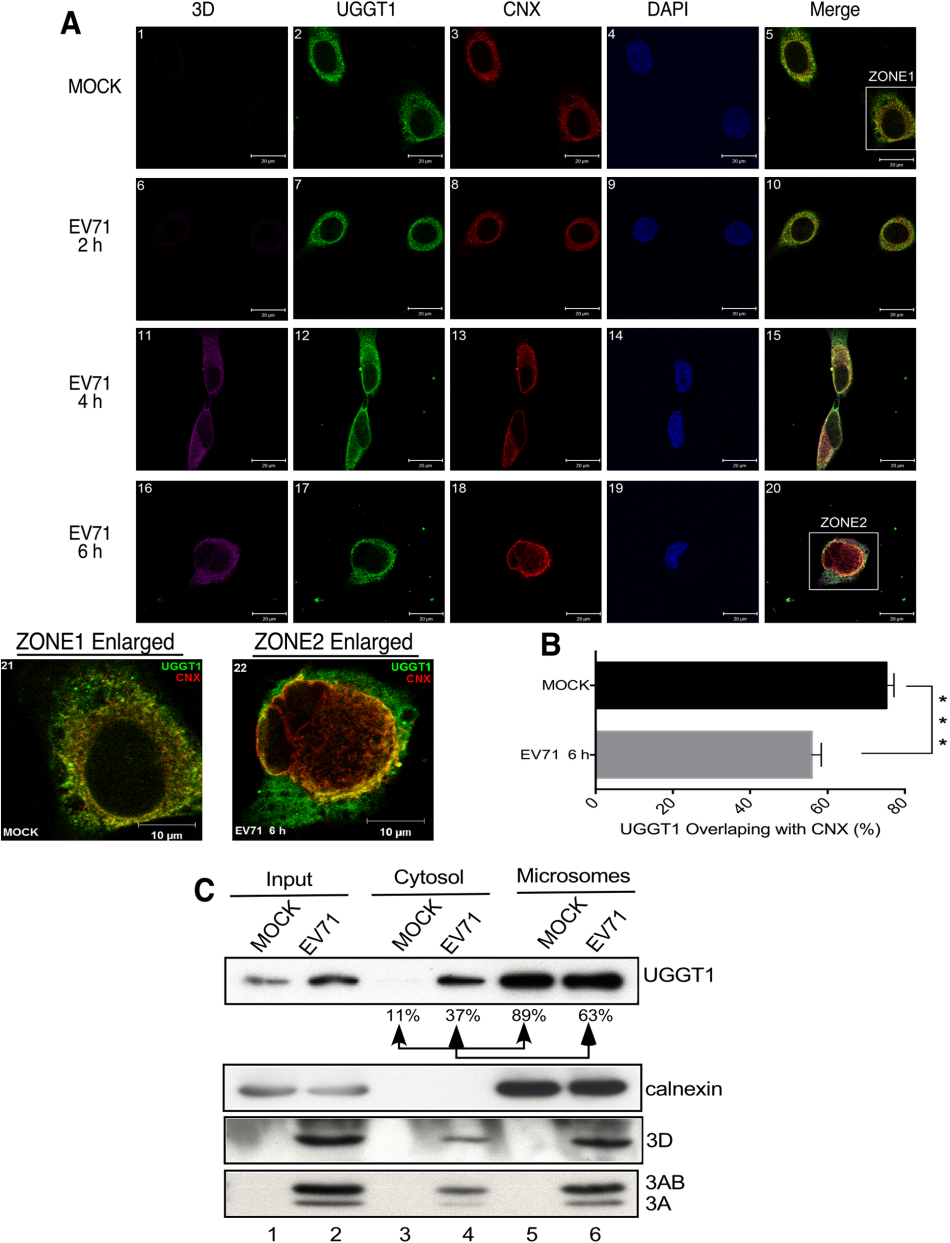
UGGT1 deploys from the ER lumen to the cytosol during EVA71 infection. (A) Mock-infected or EVA71-infected cells were fixed and stained with anti-3D, anti-UGGT1, and anti-CNX antibodies at 2, 4, and 6 h post-infection. Use of an anti-3D antibody is shown in panels 1, 6, 11, and 16, which were examined using a cy5 filter. Use of an anti-UGGT1 antibody is shown in panels 2, 7, 12, and 17, which were examined using an FITC filter. Use of an anti-CNX antibody is shown in panels 3, 8, 13, and 18, which were examined using a rhodamine filter. Panels 4, 9, 14, and 19 indicate Hoechst 33258 staining, and were examined with a DAPI filter. Panel 21 displays enlargement zone 1 from panel 5. Panel 22 displays enlargement zone 2 from panel 20. (B) Percentage of UGGT1 and CNX signal overlap as calculated with ImageJ JACoP plugins from images shown in (A). The data shown represent the average and standard deviation of ten randomly selected images. ***P < 0.001, as calculated by two-tailed unpaired Student’s t-test. (C) RD cells were infected with EVA71, harvested at 6 h post-infection, separated into cytosol and microsome fractions, and subjected to Western blot analysis using anti-UGGT1 antibody. The same blot was also probed with anti-calnexin, anti-3D, and anti-3A antibodies. The results are representative of at least three independent and reproducible experiments.

UGGT1 is a key quality control factor and protein folding sensor of the ER. To determine the localization of UGGT1 in cells following EVA71 infection, we used an anti-calnexin (CNX) antibody to evaluate proteins located in the ER in an immunoprecipitation assay. CNX is a transmembrane protein on the ER. In the absence of infection, UGGT1 and CNX were shown to colocalize in the ER (Fig 6A, panels 5 and 21), but some UGGT1 began deploying out of the ER to colocalize with the 3D viral polymerase upon EVA71 infection (Fig 6A, panels 20 and 22), to the point where little UGGT1 remained in the ER with CNX. UGGT1 and CNX signals overlapped by more than 75% in mock-infected cells, but this overlap was reduced to less than 55% in EVA71-infected cells (Fig 6B). We further performed subcellular fractionation to separate the cytosol and microsome fractions in mock- or EVA71-infected cell extracts. Microsomes are vesicle-like artifacts re-formed from pieces of the ER when cells are broken up in the laboratory, and can be separated from other cellular components by differential centrifugation. The cellular protein, calnexin, serves as a marker for the ER component in the microsome fraction. UGGT1 was predominantly located within the ER microsome in the mock cell lysates (Fig 6C, lanes 3 and 5); however, during EVA71 infection, UGGT1 was found to deploy out of the ER microsome. The proportion of UGGT1 external to the ER rose from 11% to 37% upon viral infection (Fig 6C, lane 3 and 4).

**Fig 7:**
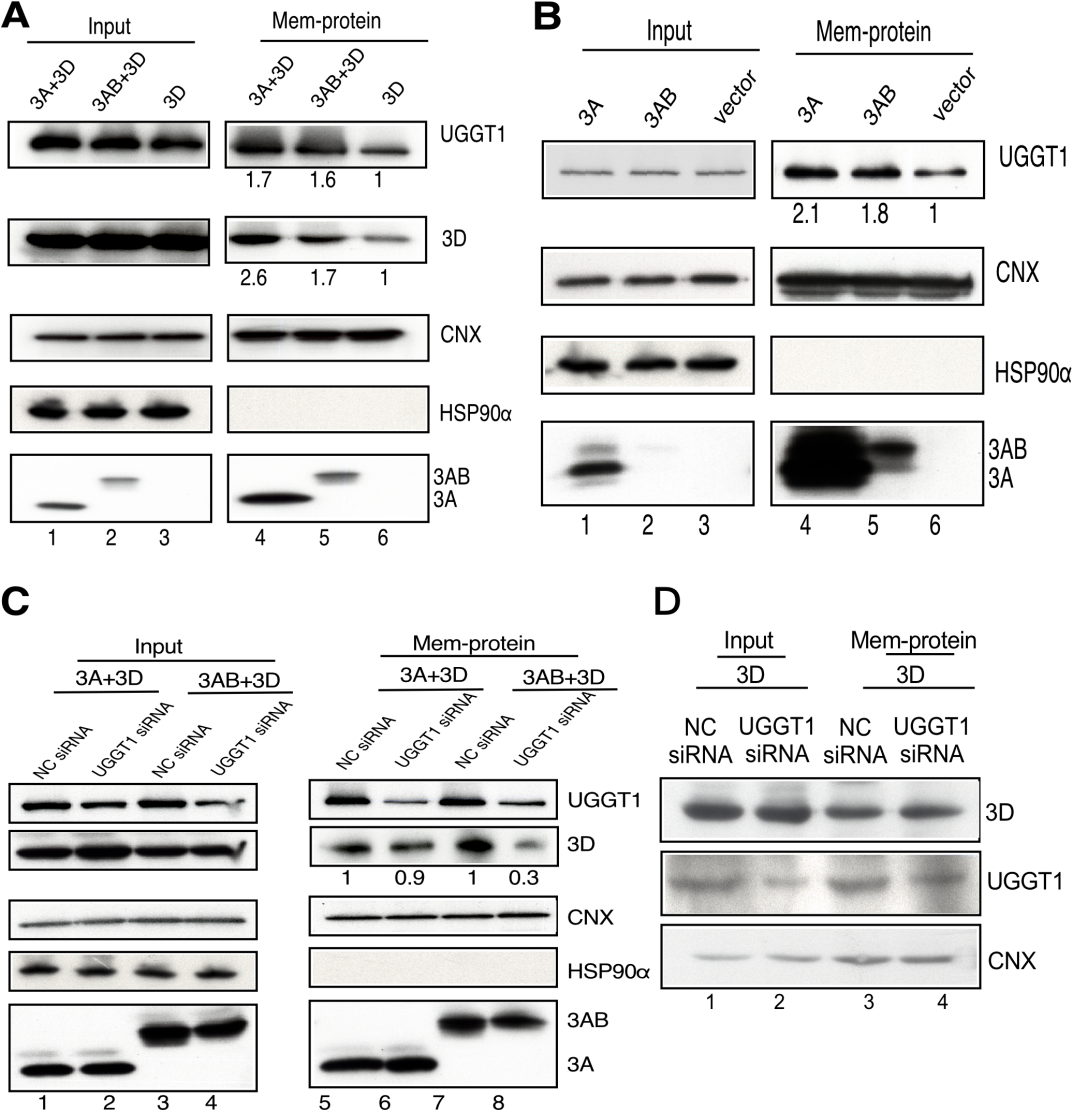
UGGT1 enhances 3D viral polymerase levels in a viral protein 3A-associated membrane fraction. (A) RD cells were co-transfected with pFLAG-3A and pFLAG-3D; pFLAG-3AB and pFLAG-3D; or pFLAG-3D only, and harvested at 48 h post-transfection. Membrane fractions were isolated and subjected to Western blot analysis. (B) Cells were transfected with pFLAG-3A, pFLAG-3AB, and pFLAG-vector and harvested at 48 h post-transfection. Membrane fractions were separated and subjected to Western blot analysis. (C) Cells were transfected with NC or UGGT1 siRNA for 48 h, and then co-transfected with pFLAG-3A and pFLAG-3D, or pFLAG-3AB and pFLAG-3D. Cells were harvested at 48 h post-transfection, and the membrane fractions were extracted and subjected to Western blot analysis. Anti-UGGT1, anti-3D and anti-3A antibodies were used. The same blot was probed with anti-CNX and anti-HSP90 antibodies. (D) Cells were transfected with NC or UGGT1 siRNA for 48 h, and then co-transfected with pFLAG-3D. Cells were harvested at 48 h post-transfection, and the membrane fractions were extracted and subjected to Western blot analysis. Anti-3D, anti-UGGT1 and anti-CNX antibodies were used. Results are representative of at least three independent experiments.

EVA71 infection induces the rearrangement of intracellular ER membranes into characteristic vesicles that assemble into viral RCs. According to Fig 1D and 1F, UGGT1 colocalizes at RCs in association with the 3D viral polymerase; however, the 3D polymerase does not possess obvious membrane-binding sequences or properties, and therefore it is unclear as to how it came to be present in RCs. In contrast, viral protein 3A contains hydrophobic domains and extensively interacts with the cellular membranes that form RCs, and thus can play an important role in membrane reorganization through its interactions with host cellular proteins. To investigate the effect of UGGT1 co-precipitation with the 3D polymerase upon RC formation, we transfected plasmids expressing viral protein 3A, 3AB, or 3D into cells, and performed the membrane protein fractionation assay. ER membranes in cells were modified by the expression of 3A and 3AB. Levels of UGGT1 and 3D polymerase in the membrane protein fraction of 3A and 3D co-expressing cells were higher than that from cells expressing 3D alone (Fig 7A). This indicates that the presence of viral proteins 3A or 3AB can enhance levels of UGGT1 and the 3D viral polymerase in the membrane protein fraction. To observe the effect of viral protein 3A upon the enhancement of UGGT1 levels in the membrane protein fraction, we used only 3A- or 3AB-expressing cells in the fractionation assay. Results showed that expression of 3A or 3AB enhanced the amount of UGGT1 by 2.1- and 1.8-fold in the membrane protein fraction (Fig 7B). To evaluate the effect of UGGT1 levels on the amount of 3D polymerase in the membrane protein fraction, we co-transfected 3A- or 3AB-expressing plasmids with pFLAG-3D into NC or UGGT1 siRNA-treated cells, and compared the amount of 3D polymerase in the membrane protein fractions. Fig 6C shows that the amount of 3D polymerase in *Uggt1* knockdown cells decreased to just 90% (3A+3D) or 30% (3AB+3D) of levels in NC siRNA-treated cells (Fig 7C). We further performed an experiment assessing 3D recruitment to cell membranes with UGGT1 knockdown in the absence of 3A or 3AB. It is well-documented in poliovirus experimental systems that 3D interacts with 3AB, and therefore it is important to ascertain whether UGGT1 directly recruits 3D, or if it merely facilitates 3D-3A interaction. In Fig 7D, the results indicated that the level of 3D recruitment to cell membranes was the same between NC siRNA- or UGGT1 siRNA-treated cells. These results show that UGGT1 can indirectly facilitate 3D-3A interactions (Fig 7D), and demonstrate that although the 3A viral protein can act to enhance levels of UGGT1 and the 3D viral polymerase in membrane protein fractions, the presence of 3D is also partly dependent on UGGT1. To the best of our understanding, UGGT1 is the first identified host protein that deploys from the ER to the cytosol following EVA71 infection, and our results indicate that UGGT1 acts to promote 3D viral polymerase levels in the viral protein 3A-associated membrane fraction, which in turn may enhance viral replication and increase viral titers.

## Discussion

Viral infection typically triggers an arms race between the virus and host cell. For example, host cells can induce UPR in the ER to restrict viral infection, but viruses can counter this by manipulating the UPR to facilitate viral propagation. In this study, we showed that expression of the key UPR factor, UGGT1, not only increases upon viral infection, but UGGT1 interaction with the EVA71 3D polymerase also has positive effects on viral growth and pathogenicity as well. Immunoprecipitation assays and MALDI-TOF analysis results indicate that the 3D viral polymerase co-precipitates with UGGT1 during EVA71 infection (Figs 1 and 2), and this interaction promotes EVA71 replication (Fig 3). Furthermore, heterozygous *uggt1* knockout mice demonstrated lower EVA71 pathogenicity than wild-type mice (Fig 4), and this may be due to reduction of positive- and negative-strand viral RNA synthesis in the absence of UGGT1 (Fig 5). We also noted that UGGT1 deploys from the ER to the cytosol upon EVA71 infection (Fig 6), where it enhances 3D polymerase levels in the membrane fraction involved in RC formation; this process is facilitated by viral protein 3A, which acts to enhance the amount of UGGT1 in the membrane fraction (Fig 7). Together, these results confirm that EVA71 can utilize the UPR host defense mechanism and the UPR factor UGGT1 to facilitate viral RNA synthesis and pathogenicity, via UGGT1 co-precipitation with the 3D viral polymerase at RCs.

We used immunoprecipitation assays to identify seven proteins that co-precipitate with the 3D polymerase, and future research could include the evaluation of other 3D polymerase-interacting host proteins as to their involvement in EVA71 replication, particularly ILF3 (Table 1). ILF3 acts to facilitate double-stranded RNA-regulated gene expression at the post-transcriptional level [50,51]. In recent years, investigators have developed an increasing interest in ILF3 and its interaction with select viral proteins [52–54]. It is known that ILF3 interacts with the 3’ stem-loop structure of dengue RNA and serves as a positive regulator of dengue virus replication [55]; however, ILF3 is also known to inhibit influenza virus replication during the early phase of infection via direct interactions with viral nucleoproteins [56]. These findings suggest that ILF3 can play both positive and negative regulatory roles in different types of viral infections. There is currently no research on the role of ILF3 in the EVA71 life cycle, and therefore further investigation on the effects of ILF3 in this respect could have significant import. Incidentally, although other proteins known to associate with viral genome RNA were also identified in Table 1, including elongation factor 2 and eukaryotic translation initiation factor 3 [57,58], our results show that RNase A treatment did not reduce the co-precipitation between UGGT1 and the 3D viral polymerase (Fig 1C), and indicate that viral genomic RNA does not mediate UGGT1-3D interaction. Further research showed that viral proteins 3C, 3AB, and 3A also co-purify with the 3D-UGGT1 complex, and may act to facilitate UGGT1 and 3D interaction.

Previous studies have found that picornavirus RNA replication occurs on the cytoplasmic surfaces of double-membrane vesicles originating from the ER, Golgi, and lysosomes in infected cells [31,59–61]. Poliovirus-induced membrane vesicles have also been linked to intracellular vesicular traffic involving COPII-dependent vesicles [62]. A recent study showed that poliovirus enriches membranes with phosphatidylinositol-4 phosphate, and promotes RNA replication through the recruitment of relevant viral and cellular proteins [37]. Our findings were similar in that UGGT1 also distributes from the ER to the cytosol to co-localize with the 3D viral polymerase, and this may help to facilitate EVA71 RC formation. To our understanding, this is the first study to report on an ER protein deploying to the cytosol to co-localize with the 3D viral polymerase. However, further research is needed to determine the exact location of UGGT1 within the viral RC membrane structure, perhaps by using an electron microscope. This research could also include further examination of the effects of UGGT1 and 3D polymerase association on the membrane secretory pathway.

In S5 Fig, we found that UGGT1 associated with JEV polymerase NS5 upon viral infection, and enhanced viral pathogenicity. However, the UGGT1 and NS5 interaction may be direct or indirect. In future, we will seek to perform additional experiments to detect other cellular or viral proteins involved in the UGGT1-NS5 complex. This research is expected to provide more information regarding the role of UGGT1 in flavivirus replication.

Mice have two UGGT genes, *Uggt1* and *Uggt2* [47], but only the *Uggt1* gene product displays reglucosylation activity, and its deletion halts reglucosylation activity in cells [45]. However, the product of *Uggt2* has no reglucosylation activity, and its function is unknown. When 80% of UGGT2 is replaced with the UGGT1 N-terminal substrate recognition domain, reglucosylation activity can be partly restored *in vitro*, demonstrating that the remaining 20% of the UGGT2 C-terminal region can serve as a functional glucosyltransferase [63]. To ascertain whether UGGT1 activity is required during viral replication, or whether UGGT1 merely acts as a protein bridge, we generated UGGT1 mutation variants lacking monoglucosylation activity, and subsequently performed UGGT1 overexpression experiments, with the results shown in S4 Fig. After comparing viral yields between cells in which UGGT1 or UGGT1(mut) was overexpressed, we found that there was no significant difference, suggesting that the enzymatic activity of UGGT1 is not required to enhance viral growth. We therefore propose that UGGT1 may primarily serve as a protein bridge that facilitates viral replication.

In summary, our results demonstrate that UGGT1 can co-precipitate with the 3D polymerase at EVA71 RCs to increase viral RNA replication. This is the first study to describe the deployment of an ER protein to the cytosol upon viral infection, and the interesting role of UGGT1 in EVA71 replication suggests that it may provide insight into the development of novel anti-EVA71 strategies. Investigators have already designed several small molecular drugs that target the 3D viral polymerase [64–67], and thus it may be feasible to develop therapies that target either the interaction between 3D and UGGT1, or between 3D and RCs. Ascertaining the functions of other cellular factors in positive-strand RNA virus replication could further facilitate the development of unique antiviral strategies, or perhaps allow the harnessing of these viral proteins for other applications.

## Materials and methods

### Ethics statement

All animal experiments were conducted in accordance with the policies and procedures set forth by the *Guide for the Care and Use of Laboratory Animals* of the National Institutes of Health. All procedures were approved by the Institutional Animal Care and Use Committee of Chang Gung University, Taiwan (IACUC approval number CGU15-017).

### Cell cultures

Human embryonal rhabdomyosarcoma (RD; from American Type Culture Collection: CRL-1620) and human glioblastoma (SF268; provided by Dr. Jim-Tong Horng lab at Chang Gung University, Taiwan) cells were maintained in Dulbecco's Modified Eagle Medium (DMEM; Gibco, Grand Island, NY) supplemented with 10% fetal bovine serum (FBS; Gibco), and cultured at 37°C in a 5% CO_2_ atmosphere. Hamster kidney fibroblast (BHK-21; from American Type Culture Collection: CCL-10) cells were maintained in RPMI 1640 Medium (Gibco, Grand Island, NY) supplemented with 2% fetal bovine serum (FBS; Gibco), and cultured at 37°C in a 5% CO_2_ atmosphere. For transfection studies, subconfluent (70%) monolayer cultures were transiently transfected or cotransfected with the respective plasmids, using Lipofectamine 2000 (Invitrogen, Carlsbad, CA). Transfected cultures were incubated for a further 48 h before being used in pull-down or co-immunoprecipitation (Co-IP) assays.

### Plasmid construction

The pFLAG-UGGT1 plasmid was constructed by amplifying UGGT1 from RD cell total RNA by RT-PCR, using a UGGT1 primer (5′-ATAAGAATGCGGCCGCGGGCTGCAAGGGAGACGCGAG-3′) containing a *Not*I restriction enzyme cutting site, and another primer (5′-GCTCTAGATCATAATTCTTCACGTTTCT-3′) containing a *Xba*I restriction enzyme site. The derived UGGT1 sequence was then cloned into a p3xFLAG-Myc-CMV vector (Sigma-Aldrich, Munich, Germany). Plasmid pHA-3D was constructed by amplifying the sequence encoding the 3D viral polymerase from the EVA71 full-length infectious cDNA clone via PCR, using an EVA71 primer (5′-CGGAATTCCGATGGGTGAGATCCAATGGAT-3′) containing a *Eco*RI restriction enzyme site and another primer (5′-ACCTCGAGATCACAATTCGAGCCAATTTC-3′) containing a *Xho*I restriction enzyme site. The derived sequence was then cloned into a pCMV-HA vector (Clontech, Palo Alto, CA).

A UGGT1 variant in which the amino acid residues critical to monoglucosylation activity, aa 1452–1457, were deleted (UGGT1(mut)), was generated by two-step overlap PCR mutagenesis. Primers *(5’-CGAGTAATAACTTCTTTGTGGA-3’) and (5’-ATGAATCATGTTAAGATTTGAAAG-3’) were used to generate the 5’ fragment and primers (5’-CTTTCAAATCTTAACATGATTCAT-3’) and *(5’- GGAATTCCGGAGACAGATCA-3’) were used to generate the 3’ fragment. Primers designated by asterisks were then used to amplify the overlapping fragments for substitution, via *Spe1* and *EcoR1* sites, into the UGGT1 expression vector (pFLAG-UGGT1) described above. The mutation was confirmed by DNA sequencing, and the resulting plasmid DNA was designated pFLAG-UGGT1(mut) [47].

### Immunoprecipitation and co-IP assays

RD cells were infected with EVA71 (strain 4643/TW/98) at a multiplicity of infection (MOI) of 10 and incubated for 6 h, prior to conducting immunoprecipitation or Co-IP assays. Infected cells were lysed in a buffer containing 50 mM Tris-HCl (pH 7.4), 150 mM sodium chloride (NaCl), 1% Triton X-100, and a protease inhibitor cocktail (Roche, Mannheim, Germany). Cell lysates were precleared with mouse immunoglobulin G (IgG) agarose and incubated with a mouse anti-3D monoclonal antibody on ice for 2 h, after which 50 μL of protein G-Sepharose beads were added, and the mixtures were incubated at 4°C overnight. Proteins bound to the beads were eluted into a 1× sodium dodecyl sulfate (SDS) running buffer by heating at 95°C for 5 min. For RNase A treatment, 100 μL of RNase A in an RNase A working buffer (0.5 U) was added before any antibodies, and the samples were incubated at 37°C for 25 min. Total degradation RNA was extracted using an RNeasy kit (Qiagen, Chatsworth, CA), according to the manufacturer's recommendations, and gel analysis was conducted. For the JEV immunoprecipitation assay, BHK-21 cells were infected with JEV (strain T1P1) and incubated for 24 h, prior to conducting the immunoprecipitation assay.

### MALDI-TOF MS analysis

Pull-down products containing eluted proteins were boiled, subjected to 8–16% sodium dodecyl sulfate polyacrylamide gel electrophoresis (SDS-PAGE), and visualized by silver staining or Western blotting. Each protein band was excised, destained, reduced, alkylated, and digested with trypsin. To extract the polypeptides, gel particles were subjected twice to consecutive 20 mM sodium bicarbonate and 5% formic acid in 50% acetonitrile treatments. The supernatants were combined and lyophilized, and the dried polypeptides were recovered by adding 10 μL of 0.1% formic acid, followed by sonication for 1 min. The recovered polypeptides were further purified using a ZipTip C18 column (Millipore, Billerica, MA), and eluted with acetonitrile to a final volume of 3 μL. Protein bands were excised and identified using in-gel trypsin digestion, then analyzed using a Bruker Ultraflex MALDI-TOF mass spectrometer (Bremen, Germany). After removing the masses derived from the standards, trypsin, matrix proteins, and keratins, the monoisotopic mass lists for each protonated peptide were subjected to database searches, and mass lists were exported to the Biotool 2.0 software package to perform peptide mass fingerprinting, using the Mascot (http://www.matrixscience.com) algorithm scoring to identify proteins.

### Immunofluorescence microscopic analysis

RD cells were seeded on 20-mm coverslips to 60% confluency and infected with EVA71 (strain 4643/TW/98) at an MOI of 10. At various post-infection timepoints, cells were washed with phosphate-buffered saline (PBS) and fixed with 4% formaldehyde for 30 min at room temperature (RT). Cells were then washed with PBS and permeabilized using 0.75% Triton X-100 for 5 min at RT, then washed again with PBS and incubated in blocking solution (PBS containing 0.5% bovine serum albumin) for 1 h at RT. Cells were then immunostained with an anti-double-strand RNA antibody, J2 (diluted 1:200; Scicons, Szirák, Hungary); an anti-3D antibody, clone 1 (diluted 1:500; prepared in the lab from recombinant 3D protein); an anti-UGGT1 antibody, K-16 (diluted 1:200; Santa Cruz Biotechnology, Santa Cruz, CA); and an anti-CNX (calnexin) antibody, H-70 (diluted 1:400; Santa Cruz Biotechnology) for 2 h at 37°C. After washing three times with PBS, cells were incubated with Alexa Fluor 568-conjugated donkey anti-goat IgG (Invitrogen) and Alexa Fluor 488 goat anti-mouse IgG (Invitrogen) for 1 h at RT. Cell nuclei were stained using Hoechst 33258 (1:500 dilution) for 20 min, according to methods previously described [68]. The cells were then observed using a confocal laser-scanning microscope (LSM 510 NLO; Zeiss, Jena, Germany).

### Transfection of UGGT1 siRNA

RD cells were cultured in six-well plates (2×10^5^ cells/well) for 24 h and then transfected with UGGT1 siRNA (UGGT1-HSS183580; Invitrogen), using Lipofectamine 2000 according to the manufacturer’s protocol (Invitrogen).

### Cell viability

The cell viability assay was performed using CellTiter-Glo Luminescent Cell Viability Assay (Promega). For the viability assays, to quantitate ATP generated by metabolically active cells, negative control (NC) or UGGT1 siRNA transfected cells were plated in 96-well plates at 5,000 cells/well. Cells were lysed with CellTiter-Glo Luminescent Cell Viability Assay reagent (Promega), and luminescence was read using the GloMax Explorer System according to the manufacturer's instructions.

### Analysis of viral RNA levels

Following transfection with NC or UGGT1 siRNA for 2 days, RD cells were seeded onto 12-well plates and incubated for 24 h. Cells were then plated to six-well plates (6×10^5^ cells/well) and infected with EVA71 at an MOI of 1. After 60 min absorption at 37°C, the cells were washed twice and supplemented with medium, then incubated at 37°C for the indicated time periods, after which intracellular RNA was extracted using an RNeasy kit (Qiagen, Chatsworth, CA). Viral RNA was detected via quantitative real-time RT-PCR with a Roche RT-PCR kit and a Lightcycler LC480 apparatus. The oligonucleotide primers and the probe for detecting EVA71 RNA were designed by Verstrepen *et al* [69]. Each sample was assayed in triplicate, and experiments were independently performed three times. The obtained data were analyzed using Roche Lightcycler LC480 system software. EVA71 RNA yields were normalized to that of actin RNA.

### Slot blotting

Slot blot analysis for detecting positive-strand and negative-strand viral RNA was performed as previously described [70]. Viral RNA was extracted and dissolved in a solution of formaldehyde and 20× SSC for 30 min at 60°C. The reaction was then loaded onto a nitrocellulose membrane in the slot blot manifold. After washing twice, the nitrocellulose membrane was removed, air dried, and UV crosslinked. Digoxigenin-labeled RNA probes of 100 ng, specific for the genome or anti-genome of EVA71, were produced using a DIG Northern starter kit (Roche). The hybridization and detection procedures were performed according to the manufacturer’s protocol.

### Enterovirus 71-Luc replicon assays

For EVA71-Luc replicon assays, RD cells were transfected with NC or UGGT1 siRNA. Three days after transfection, the EVA71-Luc replicon (kindly provided by Dr. Craig E. Cameron) was transfected into cells. After 6 h, cell lysates were collected, and luciferase expression levels were determined with the luciferase reporter assay (Promega, Madison, WI) according to the manufacturer’s instructions.

### Dicistronic or monocistronic expression assay

For the dicistronic expression assay, RD cells were transfected with UGGT1 siRNA, and after 3 days, a dicistronic construct, pRHF-EVA71, was cotransfected with UGGT1 siRNA to RD cells. After 2 days, cell lysates were prepared in a passive buffer (Promega) and examined for Renilla luciferase (RLuc) and Firefly luciferase (FLuc) activities with a Lumat LB 9507 bioluminometer (EG&G Berthold, Wildbad, Germany), using dual-luciferase reporter assays (Promega) conducted in accordance with the manufacturer’s instructions.

### Image analysis

Pixel colocalization of different color channels in confocal images was analyzed using Image J software and the ColocalizeRGB and Area Calculator plugins.

### Protein detection with western blotting

Cellular membrane fractions were collected using the Mem-PER Plus Membrane Protein Extraction Kit (ThermoFisher Scientific, San Jose, CA), in accordance with the manufacturer’s instructions. Approximately 40 μg of membrane proteins were separated with 12% polyacrylamide gels for SDS-PAGE, and electroblotted onto polyvinylidene fluoride (PVDF) membranes (BioRad, Richmond, CA). PVDF membranes were blocked for 2 h at RT in 5% milk-TBST (25 mM Tris-HCl, pH 7.5, 150 mM NaCl, 0.05% Tween 20), and then stained with anti-3D antibody (diluted 1:10,000), anti-3A antibody (diluted 1:1,000), anti-UGGT1 antibody (diluted 1:500), anti-VP2 antibody, MAB979 (diluted 1:2,000; MILLIPORE), anti-CNX (calnexin) antibody (diluted 1:1,000), and an anti-HSP90 (heat shock protein 90) antibody, ADI-SPS-771 (diluted 1:1,000; Enzo Life Sciences, Farmingdale, NY) for 2 h at 37°C. Afterwards, the membranes were washed for four times with TBST, and incubated at RT for 1 h with a peroxidase-conjugated secondary antibody (diluted 1:1,000), after which Amersham ECL Prime (GE Healthcare, Waukesha, WI) was used for chemiluminescence detection, and the signal was recorded on X-ray films.

### Subcellular fractionation

RD cells were mock infected or infected with EVA71 at an MOI of 10. The cells were harvested at 6 h post-infection. EVA71-infected cells were washed with PBS on ice, scraped into PBS, and collected by centrifugation for 5 min at 1,500 × g. Cell pellets were resuspended in hypotonic solution (42 mM KCl, 10 mM MgCl_2_, 10 mM HEPES, and pH adjusted to 7.4) and homogenized using a cell cracker (8.020-mm internal diameter, 8.010-mm bead diameter; HGM, Heidelberg, Germany). The homogenates were subjected to centrifugation for 10 min at 7,000 × g, after which mitochondrial-rich pellet was removed. The supernatant was collected and the concentration adjusted to 1 μg/μL using a hypotonic solution, then centrifuged for 45 min at 55,000 rpm at 4°C. The resulting supernatant was collected and designated as the cytosolic fraction. The pellet was resuspended in lysis buffer (10 mM HEPES, pH 7.9, 1.5 mM MgCl_2_, 10 mM KCl, 0.5 mM DTT, 1 mg/mL leupeptin, 2 mg/mL aprotonin, 1 mg/mL pepstatin A, 0.5 mM PMSF, 10 mM β-glycerophosphate, 1 mM sodium vanadate, and 0.1% Triton X-100) to derive the microsome-rich fraction.

### Virus growth and plaque assay

Following transfection with either NC or UGGT1 siRNA for 2 days, RD and SF268 cells were seeded to 12-well plates and incubated for 24 h. The cells were then infected with EVA71 (strain 4643/TW/98) or EVD68 (strain CGMH/TW/14) at an MOI of 10 or 0.1. The viruses were allowed to adsorb for 1 h at 37°C. At various timepoints post-infection, cell lysates and supernatants of the cell culture medium were collected to determine viral titers, using plaque assays. At the final time point, cell lysates were collected to measure UGGT1 expression levels. For plaque assays, virus stocks were serially diluted in PBS and allowed to adsorb onto confluent cells for 1 h at 37°C. The inoculum was then removed, and cells were washed twice with PBS and then covered with 3 mL of an agar medium. After 4 days of incubation, plaques were counted, and virus concentration was calculated as PFU/mL.

### Generation of UGGT1 knockout mice

The mouse strain used in this study was created from an ES cell clone (KOMP ID: CSD66441, EPD0550_1_E05 clone) obtained from the Knockout Mouse Project (KOMP) Repository supported by the US National Center for Research Resources-National Institutes of Health (NCRR-NIH), and which was generated by the CSD consortium for KOMP (https://www.komp.org/). The ES cell clone was used to generate chimeric mice. Germline transmitted animals were bred at the Transgenic Mouse Model Core Facility of the National Core Facility Program for Biotechnology (NCFPB), Academia Sinica, Taiwan. Animal care and handling were approved by the Institutional Animal Care and Use Committee of Chang Gung University.

### Infection of virus in mice

*Uggt1* heterozygous knockout mice were housed under specific-pathogen-free conditions in individual ventilated cages. Institutional guidelines for animal care and use were strictly followed. Mice were intraperitoneally administered with 10^5^ PFU/mouse of EVA71 strain MP4 [46], and were then monitored daily for pathological signs, and sacrificed at various times post-inoculation. The severity of central nervous system (CNS) syndromes was scored from 0 to 4 using the following criteria for scoring CNS diseases: 4 = death, 3 = paralysis of both hind legs, 2 = paralysis of one hind leg, 1 = jerky movement, and 0 = normal movement. In the JEV-infected suckling mice model, 1-day-old WT or Uggt1 heterozygous knockout mice were injected with 10^4^ PFU/mouse of JEV strain T1P1, and on Day 7 after infection, JEV was extracted from brain tissues and quantitated.

### Determination of viral titers in infected mice

The tissues and organs of EVA71-infected mice and JEV-infected mice were harvested and stored at −80°C, and homogenized in DMEM on ice using a Precellys 24 (Bertin Technologies, Montigny, France) homogenizer. Viral titers in the supernatants of clarified homogenates were determined by a plaque assay as described above, and expressed as virus titers (PFU/ml).

## Supporting information

S1 Fig Mass spectrometry analysis results for UGGT1. (A) Mass spectrometry results were used to query the NCBI human and viral protein databases, and this led to the identification of UGGT1 as one of the proteins associating with the EVA71 3D viral polymerase. The UGGT1 amino acid sequence is shown here, with matched peptides from the mass spectrometry analysis marked in bold red.
(TIF)

S2 Fig Cell proliferation and viability assay on RD cells with siRNA-reduced UGGT1 expression. (A) Western blot depicting the knockdown of UGGT1 with siRNA as indicated. (B) Proliferation of NC siRNA- or UGGT1 siRNA-transfected cells was analyzed by counting cell numbers every 24 h. (C) Results of the CellTiter-Glo Luminescent Cell Viability Assay (Promega) following transfection with the indicated siRNAs. Error bars indicate the standard deviation (SD) of three replicates.
(TIF)

S3 Fig UGGT1 promotes EVA71 and EVD68 replication and propagation. (A) and (B) EVA71 propagation in SF268 cells treated with NC or UGGT1 siRNA for 48 h and then infected with EVA71 at an MOI of 10 or 0.1. Plaque assays were conducted at the indicated timepoints after infection to measure viral titers. The left panels provide evidence of Uggt1 knockdown following siRNA treatment. (C) and (D) RD cells were transfected with 1, 2, or 4 μg of pFlag-UGGT1 or pFlag-vector, then infected 48 h later with EVA71 at an MOI of 10. Plaque assays were performed to measure viral yields at 4 and 6 h post-infection. (E) and (F) EVD68 propagation in RD cells treated with NC or UGGT1 siRNA for 48 h and then infected with EVD68 at an MOI of 10 or 0.1. Viral titers were measured by plaque assays at the indicated time points post-infection, and the left panels confirm Uggt1 knockdown following siRNA treatment. ***P < 0.001, **P < 0.01, and *P < 0.05, as calculated by two-tailed unpaired Student’s t-test.
(TIF)

S4 Fig EVA71 viral yield in UGGT1 or UGGT1(mut) overexpressed cells. (A) RD cells were transfected with 4 μg of pFLAG-vector or pFLAG-UGGT1 or pFLAG-UGGT1(mut) for 48 h, and then challenged with EVA71 at an MOI of 10. A plaque assay was performed to measure viral yields at 6 h post-infection. (B) UGGT1 was overexpressed by respectively transfecting 4 μg of plasmid pFLAG-vector or pFLAG-UGGT1 or pFLAG-UGGT1(mut) to RD cells, and the panels show the corresponding increase in UGGT1 levels following overexpression, with actin serving as a loading control.
(TIF)

S5 Fig UGGT1 associates with JEV polymerase NS5 and promotes JEV pathogenicity in suckling mice. (A) JEV-infected and mock-infected cells were harvested and subjected to immunoprecipitation assays. Immunoprecipitation assays were performed using an anti-UGGT1 antibody, and the precipitates were analyzed by Western blotting using an anti-NS5 antibody. (B) 1-day-old WT or Uggt1 heterozygous knockout mice were injected with 10^4^ PFU/mouse of JEV strain T1P1, and on Day 7 after infection, JEV was extracted from brain tissues and quantitated. ***P < 0.001 as calculated by two-tailed unpaired Student’s t-test.
(TIF)

S6 Fig EVA71 IRES activity in NC or UGGT1 siRNA-transfected cells. (A) RD cells were transfected with NC siRNA or UGGT1 siRNA for 48 h, after which the dicistronic construct, pRHF-EVA71, was transfected. After 48 h, the Renilla luciferase (RLuc) and FLuc activity in cell lysates was analyzed. Bars in the histogram represent FLuc/RLuc activity percentages. Experiments were performed in triplicate to derive the bar graph.
(TIF)

## References

[1] Salonen A, Ahola T, Kaariainen L (2005) Viral RNA replication in association with cellular membranes. Curr Top Microbiol Immunol 285: 139–173. 15609503 10.1007/3-540-26764-6_5 PMC7120253

[2] Belov GA, van Kuppeveld FJ (2012) (+)RNA viruses rewire cellular pathways to build replication organelles. Curr Opin Virol 2: 740–747. doi: 10.1016/j.coviro.2012.09.006 23036609 10.1016/j.coviro.2012.09.006 PMC7102821

[3] Koonin EV, Dolja VV (1993) Evolution and taxonomy of positive-strand RNA viruses: implications of comparative analysis of amino acid sequences. Crit Rev Biochem Mol Biol 28: 375–430. doi: 10.3109/10409239309078440 8269709 10.3109/10409239309078440

[4] Koonin EV, Wolf YI, Nagasaki K, Dolja VV (2008) The Big Bang of picorna-like virus evolution antedates the radiation of eukaryotic supergroups. Nat Rev Microbiol 6: 925–939. doi: 10.1038/nrmicro2030 18997823 10.1038/nrmicro2030

[5] McMinn PC (2002) An overview of the evolution of enterovirus 71 and its clinical and public health significance. FEMS Microbiol Rev 26: 91–107. 12007645 10.1111/j.1574-6976.2002.tb00601.x

[6] Schmidt NJ, Lennette EH, Ho HH (1974) An apparently new enterovirus isolated from patients with disease of the central nervous system. J Infect Dis 129: 304–309. 4361245 10.1093/infdis/129.3.304

[7] Chumakov M, Voroshilova M, Shindarov L, Lavrova I, Gracheva L, et al (1979) Enterovirus 71 isolated from cases of epidemic poliomyelitis-like disease in Bulgaria. Arch Virol 60: 329–340. 228639 10.1007/BF01317504

[8] Nagy G, Takatsy S, Kukan E, Mihaly I, Domok I (1982) Virological diagnosis of enterovirus type 71 infections: experiences gained during an epidemic of acute CNS diseases in Hungary in 1978. Arch Virol 71: 217–227. 6285858 10.1007/BF01314873

[9] Shindarov LM, Chumakov MP, Voroshilova MK, Bojinov S, Vasilenko SM, et al (1979) Epidemiological, clinical, and pathomorphological characteristics of epidemic poliomyelitis-like disease caused by enterovirus 71. J Hyg Epidemiol Microbiol Immunol 23: 284–295. 231067

[10] Huang PN, Shih SR (2014) Update on enterovirus 71 infection. Curr Opin Virol 5: 98–104. doi: 10.1016/j.coviro.2014.03.007 24727707 10.1016/j.coviro.2014.03.007

[11] Lum LC, Wong KT, Lam SK, Chua KB, Goh AY, et al (1998) Fatal enterovirus 71 encephalomyelitis. J Pediatr 133: 795–798. 9842048 10.1016/s0022-3476(98)70155-6

[12] Ho M, Chen ER, Hsu KH, Twu SJ, Chen KT, et al (1999) An epidemic of enterovirus 71 infection in Taiwan. Taiwan Enterovirus Epidemic Working Group. N Engl J Med 341: 929–935. doi: 10.1056/NEJM199909233411301 10498487 10.1056/NEJM199909233411301

[13] Chan KP, Goh KT, Chong CY, Teo ES, Lau G, et al (2003) Epidemic hand, foot and mouth disease caused by human enterovirus 71, Singapore. Emerg Infect Dis 9: 78–85. doi: 10.3201/eid1301.020112 12533285 10.3201/eid1301.020112 PMC2873753

[14] Shimizu H, Utama A, Onnimala N, Li C, Li-Bi Z, et al (2004) Molecular epidemiology of enterovirus 71 infection in the Western Pacific Region. Pediatr Int 46: 231–235. doi: 10.1046/j.1442-200x.2004.01868.x 15056257 10.1046/j.1442-200x.2004.01868.x

[15] Zhang Y, Tan XJ, Wang HY, Yan DM, Zhu SL, et al (2009) An outbreak of hand, foot, and mouth disease associated with subgenotype C4 of human enterovirus 71 in Shandong, China. J Clin Virol 44: 262–267. doi: 10.1016/j.jcv.2009.02.002 19269888 10.1016/j.jcv.2009.02.002

[16] Zhang Y, Zhu Z, Yang W, Ren J, Tan X, et al (2010) An emerging recombinant human enterovirus 71 responsible for the 2008 outbreak of hand foot and mouth disease in Fuyang city of China. Virol J 7: 94 doi: 10.1186/1743-422X-7-94 20459851 10.1186/1743-422X-7-94 PMC2885340

[17] Yang F, Ren L, Xiong Z, Li J, Xiao Y, et al (2009) Enterovirus 71 outbreak in the People's Republic of China in 2008. J Clin Microbiol 47: 2351–2352. doi: 10.1128/JCM.00563-09 19439545 10.1128/JCM.00563-09 PMC2708525

[18] Chan YF, Sam IC, AbuBakar S (2010) Phylogenetic designation of enterovirus 71 genotypes and subgenotypes using complete genome sequences. Infect Genet Evol 10: 404–412. doi: 10.1016/j.meegid.2009.05.010 19465162 10.1016/j.meegid.2009.05.010

[19] Chang GH, Lin L, Luo YJ, Cai LJ, Wu XY, et al (2010) Sequence analysis of six enterovirus 71 strains with different virulences in humans. Virus Res 151: 66–73. doi: 10.1016/j.virusres.2010.04.001 20398708 10.1016/j.virusres.2010.04.001

[20] Andino R, Rieckhof GE, Achacoso PL, Baltimore D (1993) Poliovirus RNA synthesis utilizes an RNP complex formed around the 5'-end of viral RNA. EMBO J 12: 3587–3598. 8253083 10.1002/j.1460-2075.1993.tb06032.x PMC413634

[21] Thompson AA, Peersen OB (2004) Structural basis for proteolysis-dependent activation of the poliovirus RNA-dependent RNA polymerase. EMBO J 23: 3462–3471. doi: 10.1038/sj.emboj.7600357 15306852 10.1038/sj.emboj.7600357 PMC516629

[22] Marcotte LL, Wass AB, Gohara DW, Pathak HB, Arnold JJ, et al (2007) Crystal structure of poliovirus 3CD protein: virally encoded protease and precursor to the RNA-dependent RNA polymerase. J Virol 81: 3583–3596. doi: 10.1128/JVI.02306-06 17251299 10.1128/JVI.02306-06 PMC1866080

[23] Kok CC, McMinn PC (2009) Picornavirus RNA-dependent RNA polymerase. Int J Biochem Cell Biol 41: 498–502. doi: 10.1016/j.biocel.2008.03.019 18487072 10.1016/j.biocel.2008.03.019

[24] Liu YC, Kuo RL, Lin JY, Huang PN, Huang Y, et al (2014) Cytoplasmic viral RNA-dependent RNA polymerase disrupts the intracellular splicing machinery by entering the nucleus and interfering with Prp8. PLoS Pathog 10: e1004199 doi: 10.1371/journal.ppat.1004199 24968230 10.1371/journal.ppat.1004199 PMC4072778

[25] Paul AV, Peters J, Mugavero J, Yin J, van Boom JH, et al (2003) Biochemical and genetic studies of the VPg uridylylation reaction catalyzed by the RNA polymerase of poliovirus. J Virol 77: 891–904. doi: 10.1128/JVI.77.2.891-904.2003 12502805 10.1128/JVI.77.2.891-904.2003 PMC140777

[26] Liu Y, Wimmer E, Paul AV (2009) Cis-acting RNA elements in human and animal plus-strand RNA viruses. Biochim Biophys Acta 1789: 495–517. doi: 10.1016/j.bbagrm.2009.09.007 19781674 10.1016/j.bbagrm.2009.09.007 PMC2783963

[27] Pathak HB, Arnold JJ, Wiegand PN, Hargittai MR, Cameron CE (2007) Picornavirus genome replication: assembly and organization of the VPg uridylylation ribonucleoprotein (initiation) complex. J Biol Chem 282: 16202–16213. doi: 10.1074/jbc.M610608200 17392285 10.1074/jbc.M610608200 PMC2116992

[28] Shen M, Reitman ZJ, Zhao Y, Moustafa I, Wang Q, et al (2008) Picornavirus genome replication. Identification of the surface of the poliovirus (PV) 3C dimer that interacts with PV 3Dpol during VPg uridylylation and construction of a structural model for the PV 3C2-3Dpol complex. J Biol Chem 283: 875–888. doi: 10.1074/jbc.M707907200 17993457 10.1074/jbc.M707907200 PMC2186065

[29] Van Dyke TA, Flanegan JB (1980) Identification of poliovirus polypeptide P63 as a soluble RNA-dependent RNA polymerase. J Virol 35: 732–740. 6252335 10.1128/jvi.35.3.732-740.1980 PMC288867

[30] McBride AE, Schlegel A, Kirkegaard K (1996) Human protein Sam68 relocalization and interaction with poliovirus RNA polymerase in infected cells. Proc Natl Acad Sci U S A 93: 2296–2301. 8637866 10.1073/pnas.93.6.2296 PMC39789

[31] Egger D, Teterina N, Ehrenfeld E, Bienz K (2000) Formation of the poliovirus replication complex requires coupled viral translation, vesicle production, and viral RNA synthesis. J Virol 74: 6570–6580. 10864671 10.1128/jvi.74.14.6570-6580.2000 PMC112167

[32] Barco A, Carrasco L (1995) A human virus protein, poliovirus protein 2BC, induces membrane proliferation and blocks the exocytic pathway in the yeast Saccharomyces cerevisiae. EMBO J 14: 3349–3364. 7628436 10.1002/j.1460-2075.1995.tb07341.x PMC394402

[33] Suhy DA, Giddings TH Jr., Kirkegaard K (2000) Remodeling the endoplasmic reticulum by poliovirus infection and by individual viral proteins: an autophagy-like origin for virus-induced vesicles. J Virol 74: 8953–8965. 10982339 10.1128/jvi.74.19.8953-8965.2000 PMC102091

[34] Wessels E, Duijsings D, Notebaart RA, Melchers WJ, van Kuppeveld FJ (2005) A proline-rich region in the coxsackievirus 3A protein is required for the protein to inhibit endoplasmic reticulum-to-golgi transport. J Virol 79: 5163–5173. doi: 10.1128/JVI.79.8.5163-5173.2005 15795300 10.1128/JVI.79.8.5163-5173.2005 PMC1069528

[35] Dorobantu CM, van der Schaar HM, Ford LA, Strating JR, Ulferts R, et al (2014) Recruitment of PI4KIIIbeta to coxsackievirus B3 replication organelles is independent of ACBD3, GBF1, and Arf1. J Virol 88: 2725–2736. doi: 10.1128/JVI.03650-13 24352456 10.1128/JVI.03650-13 PMC3958084

[36] Arita M (2014) Phosphatidylinositol-4 kinase III beta and oxysterol-binding protein accumulate unesterified cholesterol on poliovirus-induced membrane structure. Microbiol Immunol 58: 239–256. doi: 10.1111/1348-0421.12144 24527995 10.1111/1348-0421.12144

[37] Hsu NY, Ilnytska O, Belov G, Santiana M, Chen YH, et al (2010) Viral reorganization of the secretory pathway generates distinct organelles for RNA replication. Cell 141: 799–811. doi: 10.1016/j.cell.2010.03.050 20510927 10.1016/j.cell.2010.03.050 PMC2982146

[38] Kaufman RJ (1999) Stress signaling from the lumen of the endoplasmic reticulum: coordination of gene transcriptional and translational controls. Genes Dev 13: 1211–1233. 10346810 10.1101/gad.13.10.1211

[39] Hampton RY (2000) ER stress response: getting the UPR hand on misfolded proteins. Curr Biol 10: R518–521. 10898996 10.1016/s0960-9822(00)00583-2

[40] Yoshida H, Matsui T, Hosokawa N, Kaufman RJ, Nagata K, et al (2003) A time-dependent phase shift in the mammalian unfolded protein response. Dev Cell 4: 265–271. 12586069 10.1016/s1534-5807(03)00022-4

[41] Tang WF, Yang SY, Wu BW, Jheng JR, Chen YL, et al (2007) Reticulon 3 binds the 2C protein of enterovirus 71 and is required for viral replication. J Biol Chem 282: 5888–5898. doi: 10.1074/jbc.M611145200 17182608 10.1074/jbc.M611145200

[42] Wessels E, Duijsings D, Niu TK, Neumann S, Oorschot VM, et al (2006) A viral protein that blocks Arf1-mediated COP-I assembly by inhibiting the guanine nucleotide exchange factor GBF1. Dev Cell 11: 191–201. doi: 10.1016/j.devcel.2006.06.005 16890159 10.1016/j.devcel.2006.06.005

[43] Ryder E, Gleeson D, Sethi D, Vyas S, Miklejewska E, et al (2013) Molecular characterization of mutant mouse strains generated from the EUCOMM/KOMP-CSD ES cell resource. Mamm Genome 24: 286–294. doi: 10.1007/s00335-013-9467-x 23912999 10.1007/s00335-013-9467-x PMC3745610

[44] Koudounas K, Banilas G, Michaelidis C, Demoliou C, Rigas S, et al (2015) A defence-related Olea europaea beta-glucosidase hydrolyses and activates oleuropein into a potent protein cross-linking agent. J Exp Bot 66: 2093–2106. doi: 10.1093/jxb/erv002 25697790 10.1093/jxb/erv002 PMC4669557

[45] Molinari M, Galli C, Vanoni O, Arnold SM, Kaufman RJ (2005) Persistent glycoprotein misfolding activates the glucosidase II/UGT1-driven calnexin cycle to delay aggregation and loss of folding competence. Mol Cell 20: 503–512. doi: 10.1016/j.molcel.2005.09.027 16307915 10.1016/j.molcel.2005.09.027

[46] Wang YF, Chou CT, Lei HY, Liu CC, Wang SM, et al (2004) A mouse-adapted enterovirus 71 strain causes neurological disease in mice after oral infection. J Virol 78: 7916–7924. doi: 10.1128/JVI.78.15.7916-7924.2004 15254164 10.1128/JVI.78.15.7916-7924.2004 PMC446098

[47] Arnold SM, Fessler LI, Fessler JH, Kaufman RJ (2000) Two homologues encoding human UDP-glucose:glycoprotein glucosyltransferase differ in mRNA expression and enzymatic activity. Biochemistry 39: 2149–2163. 10694380 10.1021/bi9916473

[48] Khan F (2015) Enterovirus D68: acute respiratory illness and the 2014 outbreak. Emerg Med Clin North Am 33: e19–32. 26065305 10.1016/j.emc.2014.12.011

[49] Huang PN, Lin JY, Locker N, Kung YA, Hung CT, et al (2011) Far upstream element binding protein 1 binds the internal ribosomal entry site of enterovirus 71 and enhances viral translation and viral growth. Nucleic Acids Res 39: 9633–9648. doi: 10.1093/nar/gkr682 21880596 10.1093/nar/gkr682 PMC3239202

[50] Kao PN, Chen L, Brock G, Ng J, Kenny J, et al (1994) Cloning and expression of cyclosporin A- and FK506-sensitive nuclear factor of activated T-cells: NF45 and NF90. J Biol Chem 269: 20691–20699. 7519613

[51] Ting NS, Kao PN, Chan DW, Lintott LG, Lees-Miller SP (1998) DNA-dependent protein kinase interacts with antigen receptor response element binding proteins NF90 and NF45. J Biol Chem 273: 2136–2145. 9442054 10.1074/jbc.273.4.2136

[52] Isken O, Baroth M, Grassmann CW, Weinlich S, Ostareck DH, et al (2007) Nuclear factors are involved in hepatitis C virus RNA replication. RNA 13: 1675–1692. doi: 10.1261/rna.594207 17684232 10.1261/rna.594207 PMC1986813

[53] Ruggieri A, Franco M, Gatto I, Kumar A, Rapicetta M (2007) Modulation of RANTES expression by HCV core protein in liver derived cell lines. BMC Gastroenterol 7: 21 doi: 10.1186/1471-230X-7-21 17565659 10.1186/1471-230X-7-21 PMC1913921

[54] Isken O, Grassmann CW, Sarisky RT, Kann M, Zhang S, et al (2003) Members of the NF90/NFAR protein group are involved in the life cycle of a positive-strand RNA virus. EMBO J 22: 5655–5665. doi: 10.1093/emboj/cdg562 14592965 10.1093/emboj/cdg562 PMC275419

[55] Gomila RC, Martin GW, Gehrke L (2011) NF90 binds the dengue virus RNA 3' terminus and is a positive regulator of dengue virus replication. PLoS One 6: e16687 doi: 10.1371/journal.pone.0016687 21386893 10.1371/journal.pone.0016687 PMC3046124

[56] Wang P, Song W, Mok BW, Zhao P, Qin K, et al (2009) Nuclear factor 90 negatively regulates influenza virus replication by interacting with viral nucleoprotein. J Virol 83: 7850–7861. doi: 10.1128/JVI.00735-09 19494010 10.1128/JVI.00735-09 PMC2715781

[57] Sizova DV, Kolupaeva VG, Pestova TV, Shatsky IN, Hellen CU (1998) Specific interaction of eukaryotic translation initiation factor 3 with the 5' nontranslated regions of hepatitis C virus and classical swine fever virus RNAs. J Virol 72: 4775–4782. 9573242 10.1128/jvi.72.6.4775-4782.1998 PMC110013

[58] Blackwell JL, Brinton MA (1997) Translation elongation factor-1 alpha interacts with the 3' stem-loop region of West Nile virus genomic RNA. J Virol 71: 6433–6444. 9261361 10.1128/jvi.71.9.6433-6444.1997 PMC191917

[59] Bienz K, Egger D, Troxler M, Pasamontes L (1990) Structural organization of poliovirus RNA replication is mediated by viral proteins of the P2 genomic region. J Virol 64: 1156–1163. 2154600 10.1128/jvi.64.3.1156-1163.1990 PMC249229

[60] Cho MW, Teterina N, Egger D, Bienz K, Ehrenfeld E (1994) Membrane rearrangement and vesicle induction by recombinant poliovirus 2C and 2BC in human cells. Virology 202: 129–145. doi: 10.1006/viro.1994.1329 8009827 10.1006/viro.1994.1329

[61] Schlegel A, Giddings TH Jr., Ladinsky MS, Kirkegaard K (1996) Cellular origin and ultrastructure of membranes induced during poliovirus infection. J Virol 70: 6576–6588. 8794292 10.1128/jvi.70.10.6576-6588.1996 PMC190698

[62] Rust RC, Landmann L, Gosert R, Tang BL, Hong W, et al (2001) Cellular COPII proteins are involved in production of the vesicles that form the poliovirus replication complex. J Virol 75: 9808–9818. doi: 10.1128/JVI.75.20.9808-9818.2001 11559814 10.1128/JVI.75.20.9808-9818.2001 PMC114553

[63] Arnold SM, Kaufman RJ (2003) The noncatalytic portion of human UDP-glucose: glycoprotein glucosyltransferase I confers UDP-glucose binding and transferase function to the catalytic domain. J Biol Chem 278: 43320–43328. doi: 10.1074/jbc.M305800200 12913004 10.1074/jbc.M305800200

[64] Di Marco S, Volpari C, Tomei L, Altamura S, Harper S, et al (2005) Interdomain communication in hepatitis C virus polymerase abolished by small molecule inhibitors bound to a novel allosteric site. J Biol Chem 280: 29765–29770. doi: 10.1074/jbc.M505423200 15955819 10.1074/jbc.M505423200

[65] Tedesco R, Shaw AN, Bambal R, Chai D, Concha NO, et al (2006) 3-(1,1-dioxo-2H-(1,2,4)-benzothiadiazin-3-yl)-4-hydroxy-2(1H)-quinolinones, potent inhibitors of hepatitis C virus RNA-dependent RNA polymerase. J Med Chem 49: 971–983. doi: 10.1021/jm050855s 16451063 10.1021/jm050855s

[66] Tomei L, Altamura S, Bartholomew L, Biroccio A, Ceccacci A, et al (2003) Mechanism of action and antiviral activity of benzimidazole-based allosteric inhibitors of the hepatitis C virus RNA-dependent RNA polymerase. J Virol 77: 13225–13231. doi: 10.1128/JVI.77.24.13225-13231.2003 14645579 10.1128/JVI.77.24.13225-13231.2003 PMC296079

[67] Tomei L, Altamura S, Bartholomew L, Bisbocci M, Bailey C, et al (2004) Characterization of the inhibition of hepatitis C virus RNA replication by nonnucleosides. J Virol 78: 938–946. doi: 10.1128/JVI.78.2.938-946.2004 14694125 10.1128/JVI.78.2.938-946.2004 PMC368780

[68] Weng KF, Li ML, Hung CT, Shih SR (2009) Enterovirus 71 3C protease cleaves a novel target CstF-64 and inhibits cellular polyadenylation. PLoS Pathog 5: e1000593 doi: 10.1371/journal.ppat.1000593 19779565 10.1371/journal.ppat.1000593 PMC2742901

[69] Verstrepen WA, Kuhn S, Kockx MM, Van De Vyvere ME, Mertens AH (2001) Rapid detection of enterovirus RNA in cerebrospinal fluid specimens with a novel single-tube real-time reverse transcription-PCR assay. J Clin Microbiol 39: 4093–4096. doi: 10.1128/JCM.39.11.4093-4096.2001 11682535 10.1128/JCM.39.11.4093-4096.2001 PMC88492

[70] Lin JY, Li ML, Huang PN, Chien KY, Horng JT, et al (2008) Heterogeneous nuclear ribonuclear protein K interacts with the enterovirus 71 5' untranslated region and participates in virus replication. J Gen Virol 89: 2540–2549. doi: 10.1099/vir.0.2008/003673-0 18796723 10.1099/vir.0.2008/003673-0

