# Supplementary figures and images for: UGGT1 enhances enterovirus 71 pathogenicity by promoting viral RNA synthesis and viral replication
Source: PLoS Pathog. 2017 May 17;13(5):e1006375. doi: 10.1371/journal.ppat.1006375 (PMC5435352; doi:10.1371/journal.ppat.1006375)

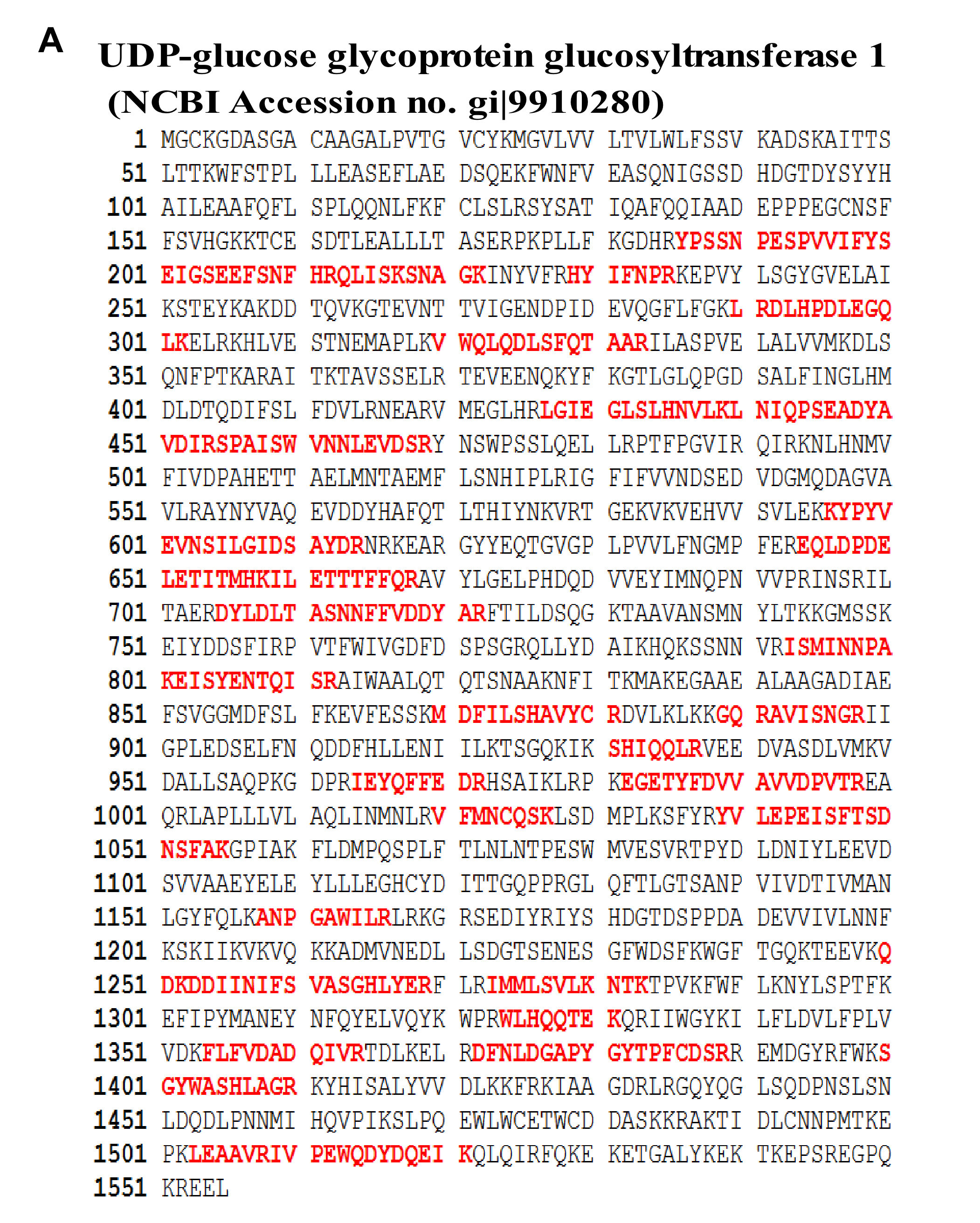

Supplement: S1 Fig — (A) Mass spectrometry results were used to query the NCBI human and viral protein databases, and this led to the identification of UGGT1 as one of the proteins associating with the EVA71 3D viral polymerase. The UGGT1 amino acid sequence is shown here, with matched peptides from the mass spectrometry analysis marked in bold red. (TIF) [file ppat.1006375.s001.tif]

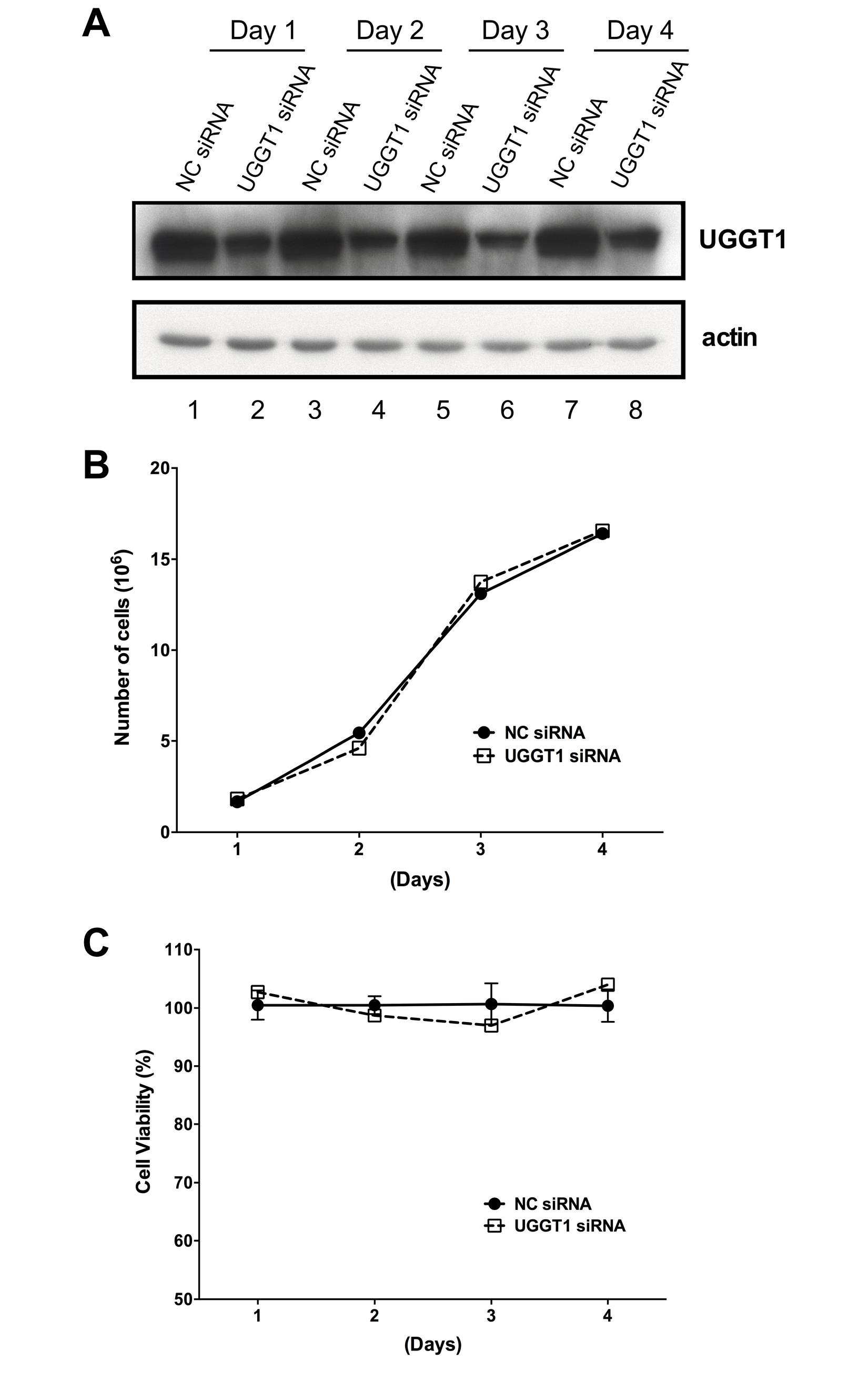

Supplement: S2 Fig — (A) Western blot depicting the knockdown of UGGT1 with siRNA as indicated. (B) Proliferation of NC siRNA- or UGGT1 siRNA-transfected cells was analyzed by counting cell numbers every 24 h. (C) Results of the CellTiter-Glo Luminescent Cell Viability Assay (Promega) following transfection with the indicated siRNAs. Error bars indicate the standard deviation (SD) of three replicates. (TIF) [file ppat.1006375.s002.tif]

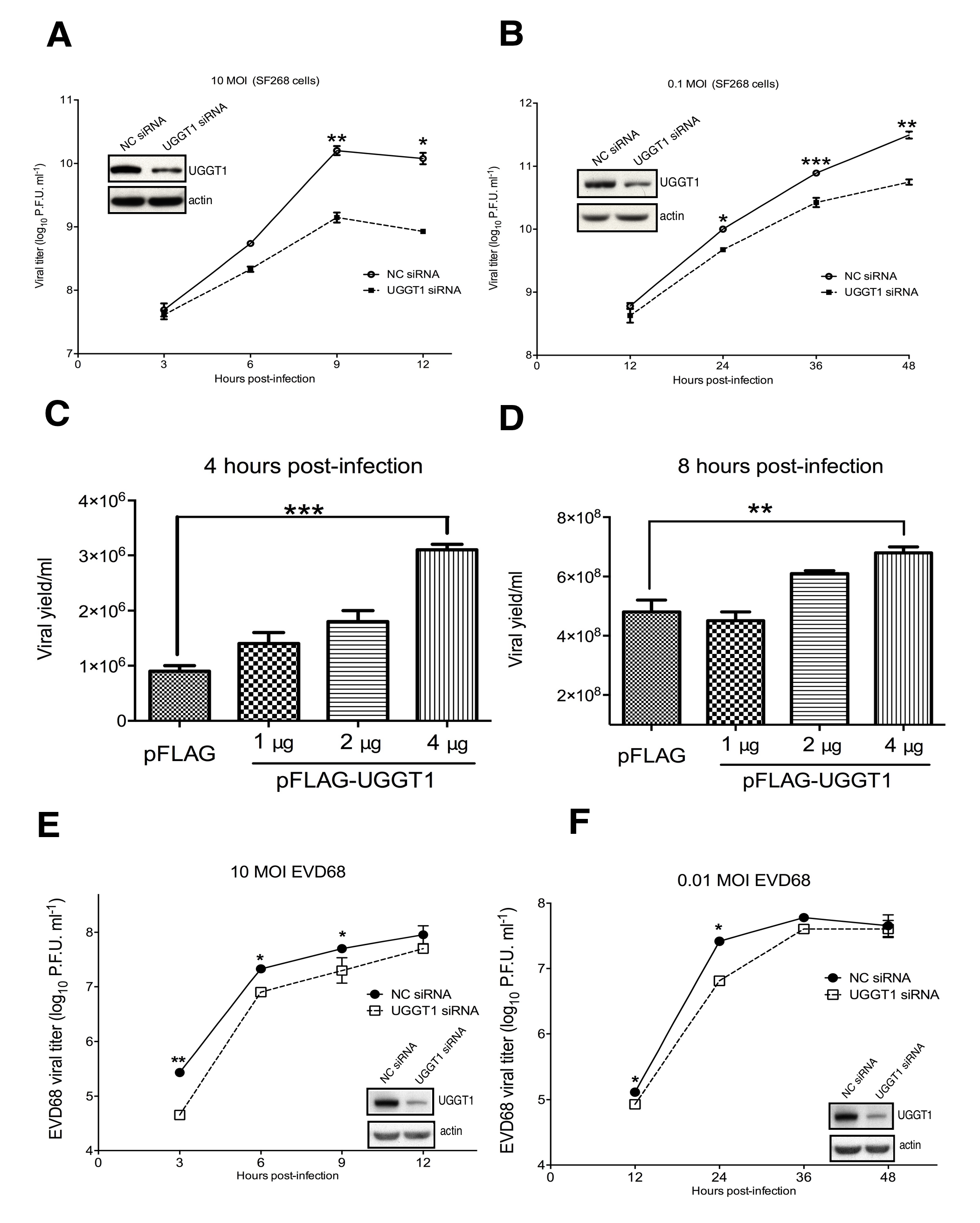

Supplement: S3 Fig — (A) and (B) EVA71 propagation in SF268 cells treated with NC or UGGT1 siRNA for 48 h and then infected with EVA71 at an MOI of 10 or 0.1. Plaque assays were conducted at the indicated timepoints after infection to measure viral titers. The left panels provide evidence of Uggt1 knockdown following siRNA treatment. (C) and (D) RD cells were transfected with 1, 2, or 4 μg of pFlag-UGGT1 or pFlag-vector, then infected 48 h later with EVA71 at an MOI of 10. Plaque assays were performed to measure viral yields at 4 and 6 h post-infection. (E) and (F) EVD68 propagation in RD cells treated with NC or UGGT1 siRNA for 48 h and then infected with EVD68 at an MOI of 10 or 0.1. Viral titers were measured by plaque assays at the indicated time points post-infection, and the left panels confirm Uggt1 knockdown following siRNA treatment. ***P < 0.001, **P < 0.01, and *P < 0.05, as calculated by two-tailed unpaired Student’s t-test. (TIF) [file ppat.1006375.s003.tif]

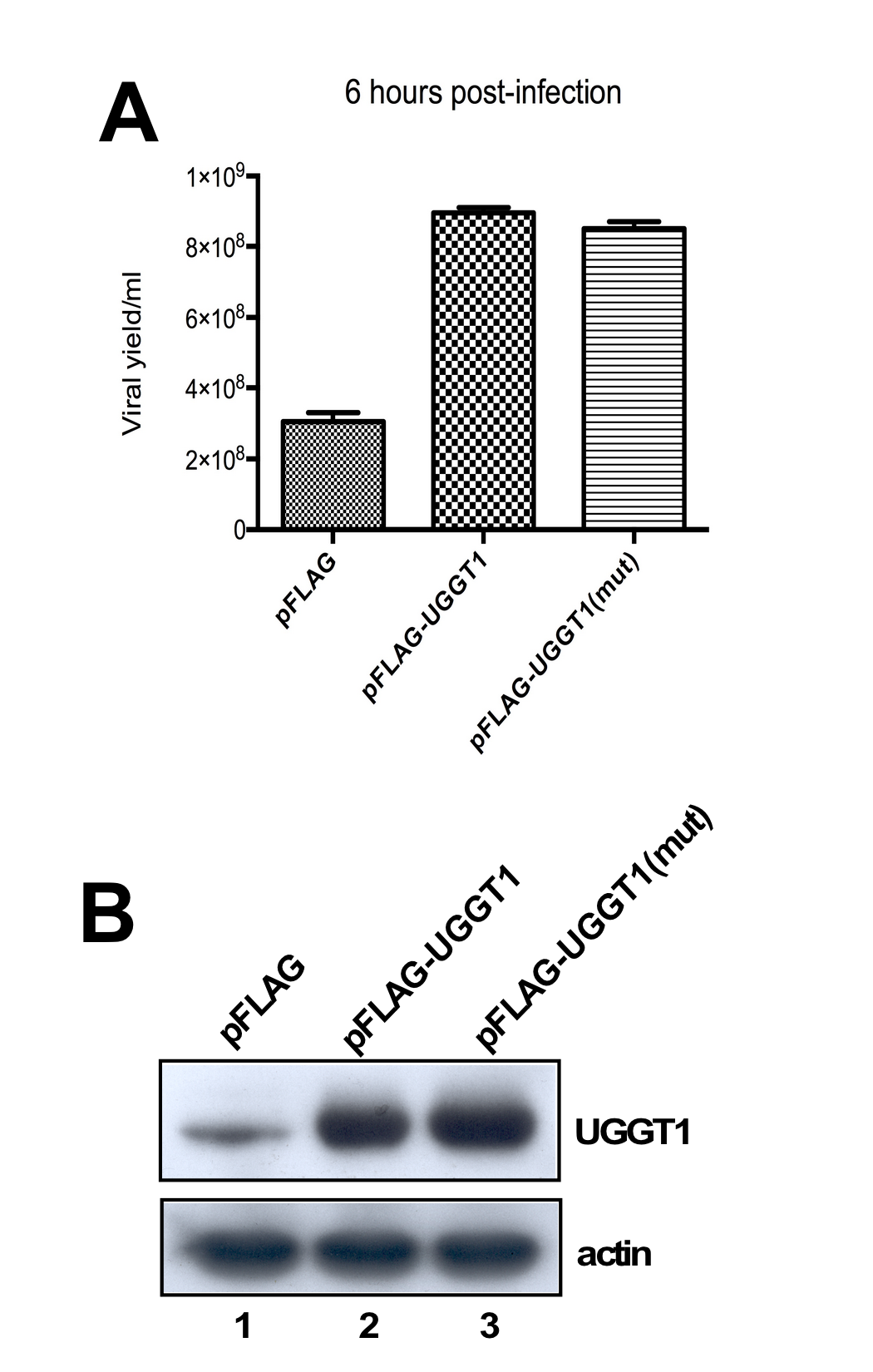

Supplement: S4 Fig — (A) RD cells were transfected with 4 μg of pFLAG-vector or pFLAG-UGGT1 or pFLAG-UGGT1(mut) for 48 h, and then challenged with EVA71 at an MOI of 10. A plaque assay was performed to measure viral yields at 6 h post-infection. (B) UGGT1 was overexpressed by respectively transfecting 4 μg of plasmid pFLAG-vector or pFLAG-UGGT1 or pFLAG-UGGT1(mut) to RD cells, and the panels show the corresponding increase in UGGT1 levels following overexpression, with actin serving as a loading control. (TIF) [file ppat.1006375.s004.tif]

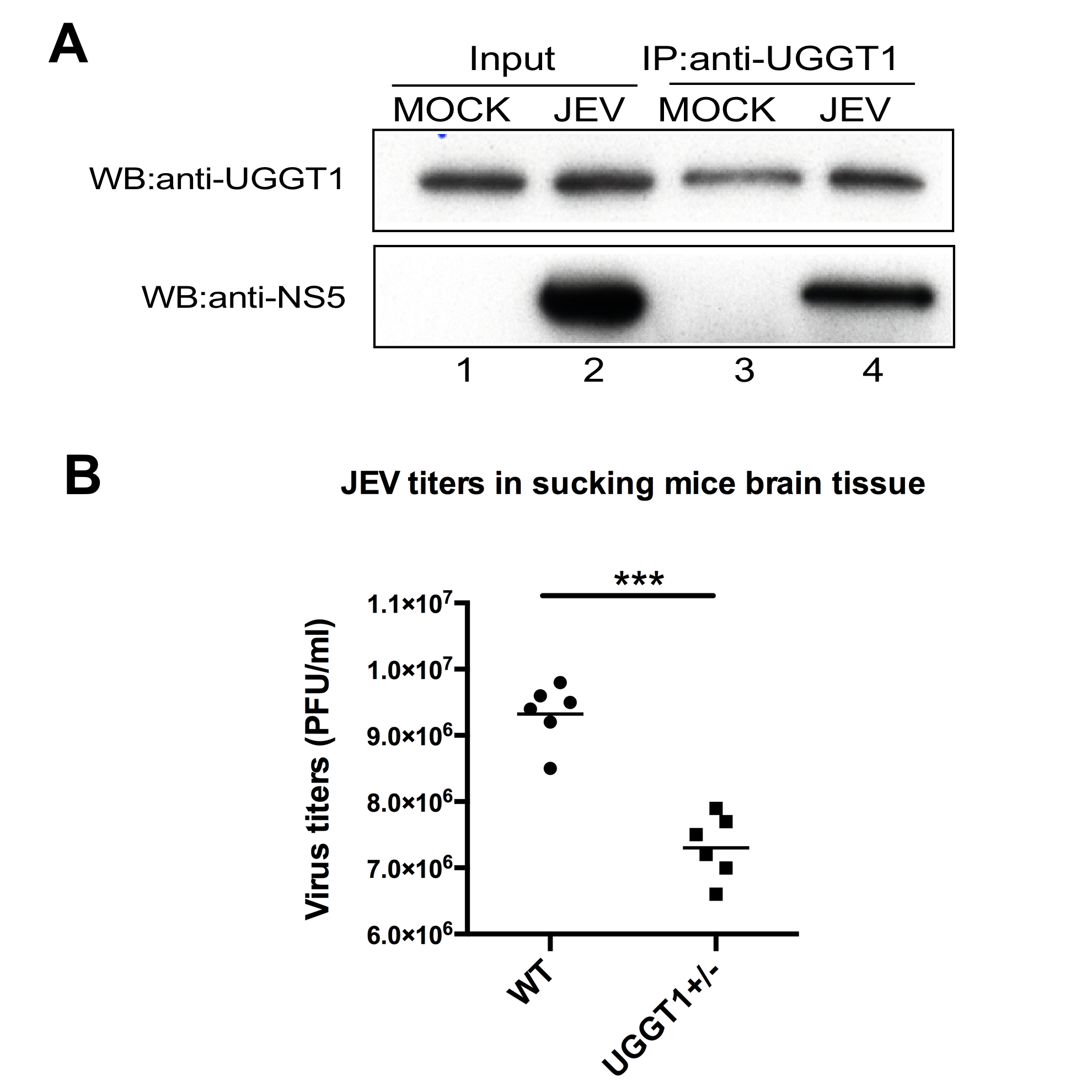

Supplement: S5 Fig — (A) JEV-infected and mock-infected cells were harvested and subjected to immunoprecipitation assays. Immunoprecipitation assays were performed using an anti-UGGT1 antibody, and the precipitates were analyzed by Western blotting using an anti-NS5 antibody. (B) 1-day-old WT or Uggt1 heterozygous knockout mice were injected with 104 PFU/mouse of JEV strain T1P1, and on Day 7 after infection, JEV was extracted from brain tissues and quantitated. ***P < 0.001 as calculated by two-tailed unpaired Student’s t-test. (TIF) [file ppat.1006375.s005.tif]

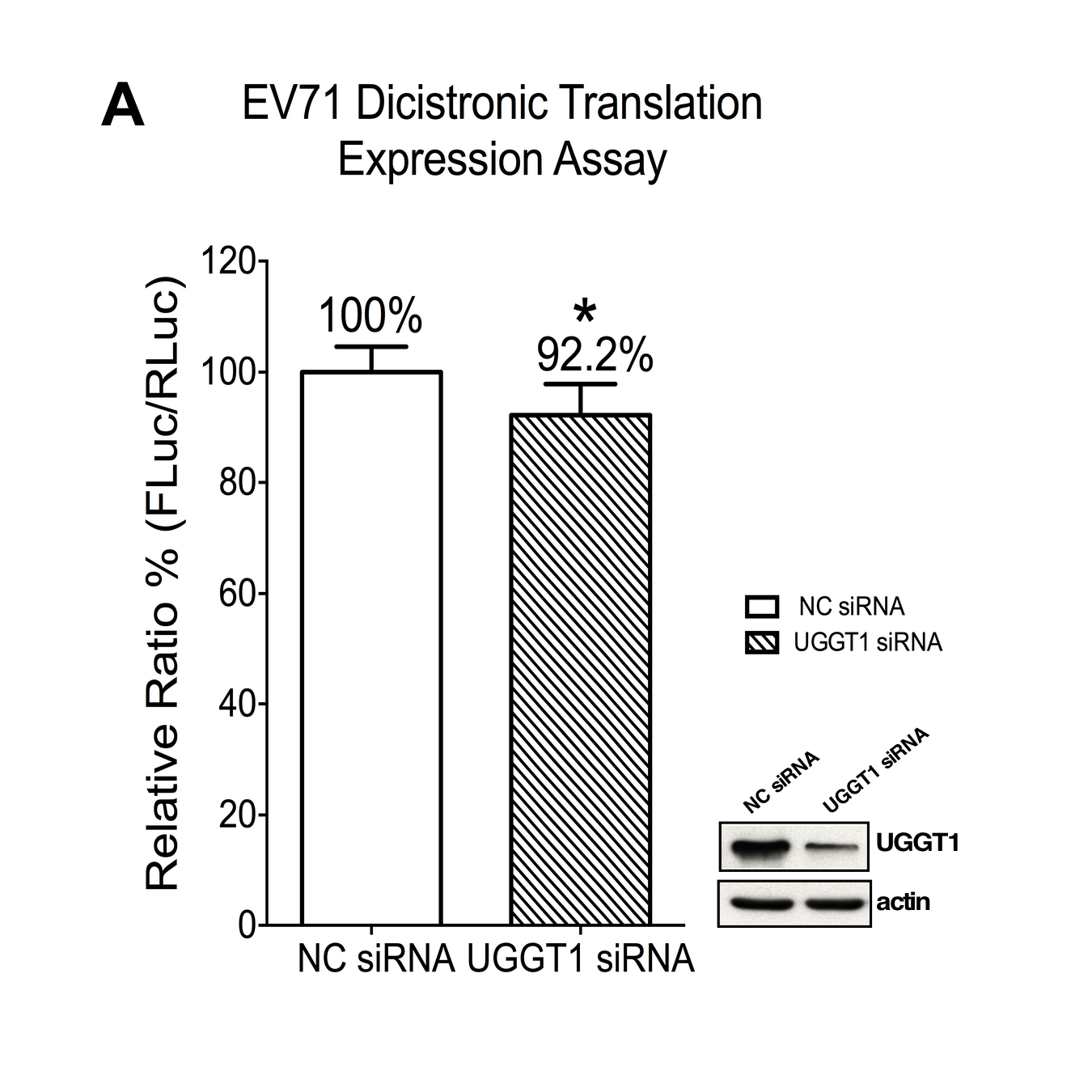

Supplement: S6 Fig — (A) RD cells were transfected with NC siRNA or UGGT1 siRNA for 48 h, after which the dicistronic construct, pRHF-EVA71, was transfected. After 48 h, the Renilla luciferase (RLuc) and FLuc activity in cell lysates was analyzed. Bars in the histogram represent FLuc/RLuc activity percentages. Experiments were performed in triplicate to derive the bar graph. (TIF) [file ppat.1006375.s006.tif]
